# Smoking Status at Diagnosis and Colorectal Cancer Prognosis According to Tumor Lymphocytic Reaction

**DOI:** 10.1093/jncics/pkaa040

**Published:** 2020-05-14

**Authors:** Kenji Fujiyoshi, Yang Chen, Koichiro Haruki, Tomotaka Ugai, Junko Kishikawa, Tsuyoshi Hamada, Li Liu, Kota Arima, Jennifer Borowsky, Juha P Väyrynen, Melissa Zhao, Mai Chan Lau, Simeng Gu, Shanshan Shi, Naohiko Akimoto, Tyler S Twombly, David A Drew, Mingyang Song, Andrew T Chan, Edward L Giovannucci, Jeffrey A Meyerhardt, Charles S Fuchs, Reiko Nishihara, Jochen K Lennerz, Marios Giannakis, Jonathan A Nowak, Xuehong Zhang, Kana Wu, Shuji Ogino

**Affiliations:** p1 Program in MPE Molecular Pathological Epidemiology, Department of Pathology, Brigham and Women’s Hospital and Harvard Medical School, Boston, MA, USA; p2 Department of Surgery, Kurume University, Kurume, Fukuoka, Japan; p3 Cancer and Translational Medicine Research Unit, Medical Research Center Oulu, Oulu University Hospital, and University of Oulu, Oulu, Finland; p4 Department of Medical Oncology, Dana-Farber Cancer Institute and Harvard Medical School, Boston, MA, USA; p5 Clinical and Translational Epidemiology Unit, Massachusetts General Hospital, and Harvard Medical School, Boston, MA, USA; p6 Division of Gastroenterology, Massachusetts General Hospital, Boston, MA, USA; p7 Department of Nutrition, Harvard T.H. Chan School of Public Health, Boston, MA, USA; p8 Channing Division of Network Medicine, Department of Medicine, Brigham and Women’s Hospital, and Harvard Medical School, Boston, MA, USA; p9 Department of Immunology and Infectious Diseases, Harvard T.H. Chan School of Public Health, Boston, MA, USA; p10 Department of Epidemiology, Harvard T.H. Chan School of Public Health, Boston, MA, USA; p11 Yale Cancer Center, New Haven, CT, USA; p12 Department of Medicine, Yale School of Medicine, New Haven, CT, USA; p13 Smilow Cancer Hospital, New Haven, CT, USA; p14 Department of Biostatistics, Harvard T.H. Chan School of Public Health, Boston, MA, USA; p15 Department of Pathology, Massachusetts General Hospital, and Harvard Medical School, Boston, MA, USA; p16 Broad Institute of MIT and Harvard, Cambridge, MA, USA; p17 Department of Medicine, Brigham and Women’s Hospital and Harvard Medical School, Boston, MA, USA; p18 Cancer Immunology and Cancer Epidemiology Programs, Dana-Farber Harvard Cancer Center, Boston, MA, USA

## Abstract

**Background:**

Smoking has been associated with worse colorectal cancer patient survival and may potentially suppress the immune response in the tumor microenvironment. We hypothesized that the prognostic association of smoking behavior at colorectal cancer diagnosis might differ by lymphocytic reaction patterns in cancer tissue.

**Methods:**

Using 1474 colon and rectal cancer patients within 2 large prospective cohort studies (Nurses’ Health Study and Health Professionals Follow-up Study), we characterized 4 patterns of histopathologic lymphocytic reaction, including tumor-infiltrating lymphocytes (TILs), intratumoral periglandular reaction, peritumoral lymphocytic reaction, and Crohn’s-like lymphoid reaction. Using covariate data of 4420 incident colorectal cancer patients in total, an inverse probability weighted multivariable Cox proportional hazards regression model was conducted to adjust for selection bias due to tissue availability and potential confounders, including tumor differentiation, disease stage, microsatellite instability status, CpG island methylator phenotype, long interspersed nucleotide element-1 methylation, and *KRAS*, *BRAF*, and *PIK3CA* mutations.

**Results:**

The prognostic association of smoking status at diagnosis differed by TIL status. Compared with never smokers, the multivariable-adjusted colorectal cancer–specific mortality hazard ratio for current smokers was 1.50 (95% confidence interval = 1.10 to 2.06) in tumors with negative or low TIL and 0.43 (95% confidence interval = 0.16 to 1.12) in tumors with intermediate or high TIL (2-sided *P*_interaction_ = .009). No statistically significant interactions were observed in the other patterns of lymphocytic reaction.

**Conclusions:**

The association of smoking status at diagnosis with colorectal cancer mortality may be stronger for carcinomas with negative or low TIL, suggesting a potential interplay of smoking and lymphocytic reaction in the colorectal cancer microenvironment.

Cigarette smoking is an established risk factor for incidence of colon and rectal cancer (1). Current smoking has appeared to be a modest risk factor for colorectal cancer patient survival (2–5). Accumulating evidence indicates that smoking influences both innate and adaptive immunity. Cigarette smoke contains thousands of harmful chemicals that may potentially suppress immune cell function, thereby promoting tumor evolution (6,7). Considering evidence for the influence of smoking on the tumor immune microenvironment, we hypothesized that smoking might influence tumor progression differentially by the degree of antitumor immune response.

A variety of endogenous and exogenous factors may exert effects on the host immune response to colorectal cancer (8–11). Tumor-infiltrating lymphocytes (TIL) and T cells have been considered as indicators of the host immune response against tumor and an attractive target for immunotherapy (10,12,13). The abundance of these cells in the tumor has been associated with longer survival in colorectal cancer patients independently of stage and microsatellite instability (MSI) status (12,14,15). Recently, we reported that the incidence risk of colorectal cancer increased by smoking was stronger for tumors with lower T-cell response, suggesting a suppression effect of smoking on T-cell–mediated immunity and an important interaction of smoking and immunity in colorectal carcinogenesis (16). Immune response in the tumor microenvironment has a crucial role in suppressing tumor progression, contributing to better patient prognosis. Considering the role of immune cells, including lymphocytes, we hypothesized that smoking status at diagnosis might be associated with higher mortality in the tumors with weaker lymphocytic reactions compared with those with stronger lymphocytic reactions.

To test our hypothesis, we used 2 large US nationwide prospective cohort studies with covariate data of 4420 colorectal cancer patients and a molecular pathological epidemiology database of 1474 patients. This comprehensive dataset enabled us to examine the prognostic association of smoking status at diagnosis according to lymphocytic reaction in the colorectal cancer tissue.

## Materials and Methods

### Study Population

We collected data from 2 prospective cohort studies in the United States: the Nurses’ Health Study (NHS, 121 701 women followed since 1976) and the Health Professionals Follow-up Study (HPFS, 51 529 men followed since 1986) (17). In both cohorts, questionnaires have been sent to participants to update information on smoking status, other lifestyle factors, and medical history every 2 years. We used the National Death Index to confirm deaths of study participants and identify unreported lethal colorectal cancer patients.

We included 1474 patients with available data on smoking exposure at diagnosis and immune profiles including lymphocytic reaction in colorectal cancer tissue. We included both colon and rectal carcinomas based on the colorectal continuum model (18). Patients were followed-up until death or the end of follow-up (January 1, 2014, for HPFS; May 31, 2014, for NHS), whichever came first. We used the inverse probability weighting (IPW) method (19,20) and covariate data of 4420 incident colorectal cancer patients to adjust for selection bias in the 1474 patients. Previous studies using IPW in our dataset showed that results with and without IPW generated similar data (19). Study physicians reviewed medical records associated with colorectal cancer diagnoses and identified cause of death for deceased participants based on medical records and death certificates. For nonresponders who had died of colorectal cancer, we obtained permission from the next of kin and reviewed medical records to gather data on date of diagnosis, stage, tumor location, and tumor grade. Formalin-fixed paraffin-embedded tissue blocks were collected from hospitals across the United States where colorectal cancer patients underwent their primary tumor resection. A study pathologist (S.O.) blinded to other data conducted a centralized review of hematoxylin and eosin–stained tissue sections of all colorectal carcinoma patients and collected data on pathological features (21). Tumor differentiation was categorized as moderate (>50% glandular area) or poor (≤50% glandular area).

Informed consent was obtained from all participants in this analysis. The study procedures and protocols were approved by the institutional review boards at the Harvard T.H. Chan School of Public Health, Brigham and Women’s Hospital (Boston, MA), and those of participating registries as required.

### Assessment of Smoking Status

Data on smoking status were collected in the 2 cohorts as reported previously (22). Current smoking status and the number of cigarettes smoked per day were reported by participants on biennial questionnaires since 1980 (NHS) and 1986 (HPFS). In the baseline questionnaires (1976 in NHS and 1986 in HPFS), participants were asked to report the age at which they began and ceased smoking (for past smokers) and the average daily consumption of cigarettes. Smoking status at colorectal cancer diagnosis was derived from the latest available questionnaire before diagnosis. Smoking status after diagnosis was derived from the available questionnaire at least more than 6 months after colorectal cancer diagnosis. We calculated cumulative pack-years of cigarettes ([cumulative average of packs per day] multiplied by [the number of years during which smoking occurred]) and duration of smoking cessation for past and current smokers (22).

### Assessment of Tumor Immunity Status

Four components of lymphocytic reaction to tumors, including TIL, intratumoral periglandular reaction, peritumoral lymphocytic reaction, and Crohn’s-like lymphoid reaction, were histopathologically evaluated as previously reported (21). Briefly, the 4 lymphocytic reaction components were scored as 0, 1+, 2+, and 3+ and graded as negative or low (0), intermediate (1+), or high (2+, 3+) by a study pathologist (S.O.) based on centralized review of hematoxylin and eosin tissue sections. Review of 398 selected patients between 2 independent pathologists (S.O. and J.N. Glickman) showed good concordance on grading of histopathologic features, including lymphocytic reaction to tumor (21).

### Analyses of Tumor Molecular Characteristics

Genomic DNA was extracted from formalin-fixed paraffin-embedded colorectal carcinoma tissue. MSI status was determined by polymerase chain reaction of 10 microsatellite markers (D2S123, D5S346, D17S250, BAT25, BAT26, BAT40, D18S55, D18S56, D18S67, and D18S487); MSI-high was defined as the presence of instability in at least 30% of the markers (18,23). Methylation status of 8 CpG island methylator phenotype (CIMP)-specific promoters (*CACNA1G*, *CDKN2A*, *CRABP1*, *IGF2*, *MLH1*, *NEUROG1*, *RUNX3*, and *SOCS1*) (24) was determined by MethyLight assay using bisulfite-treated DNA (25). CIMP-high was defined as at least 6 methylated promoters of 8 promoters, and CIMP-low or negative as 0 to 5 methylated promoters (24). Methylation levels at long-interspersed nucleotide element-1 were measured by pyrosequencing using bisulfite-treated DNA (26). Polymerase chain reaction and pyrosequencing were performed for *KRAS* (codons 12, 13, 61, and 146), *BRAF* (codon 600), and *PIK3CA* (exons 9 and 20) (27–29).

### Statistical Analyses

All statistical analyses were performed using SAS software (version 9.4, SAS Institute, Cary, NC). All *P* values were 2-sided. Our primary hypothesis test was an assessment of a statistical interaction between smoking status at diagnosis (ordinal; never, past, and current) and lymphocytic reaction in tumor tissue (binary classification of negative/low and intermediate/high) in a multivariable Cox proportional hazards regression model using the Wald test on the cross-product. We used the 2-sided α-level of .005 (30). The hazard ratio (HR) for smoking status at diagnosis in strata of lymphocytic reaction components using a reparameterization of the interaction term was also assessed in a single regression model (19).

Primary outcome endpoint of this study was colorectal cancer–specific mortality and the secondary endpoint was overall mortality. For colorectal cancer–specific survival analyses, deaths of other causes and patients with missing data on cause of death were censored. Survival time was defined as the period from diagnosis of colorectal cancer to death or the end of follow-up, whichever came first.

In all survival analyses, we used covariate data of 4420 incident colorectal cancer patients and the IPW method (Supplementary Methods available online) to reduce the selection bias due to the availability of tumor tissue (19,20). The probability of the availability of tumor tissue for each patient was estimated using a multivariable logistic regression model, and each patient with complete data was weighted by the inverse of the probability (19,20). The IPW-adjusted Kaplan-Meier method was used to estimate the distribution of colorectal cancer–specific and overall survivals, and the weighted log-rank test was performed (31). Similar results were obtained by Cox regression analyses without the IPW.

IPW-adjusted, multivariable Cox proportional hazards regression models were used to adjust for potential confounders and initially included the following: sex (ie, cohort), age at diagnosis, year of diagnosis, family history of colorectal cancer, body mass index at diagnosis, alcohol consumption at diagnosis; physical activity at diagnosis; processed meat intake at diagnosis; total fiber intake at diagnosis; tumor location; tumor differentiation; disease stage; MSI status; CIMP; long-interspersed nucleotide element-1 methylation level; and *KRAS*, *BRAF*, and *PIK3CA* mutations. A backward elimination with a threshold of *P* = .05 was performed to select variables for the final models. The proportionality of hazards assumption was assessed using a time-varying covariate, which is an interaction term of survival time and smoking status at diagnosis. The proportionality of hazards assumption was generally satisfied for cancer-specific survival (*P* > .28). All statistical tests were 2-sided.

## Results

We included 1474 colorectal cancer patients with available data on smoking status at diagnosis and lymphocytic reaction among 4420 incident colorectal cancer patients in the 2 prospective cohort studies (Table 1). The frequency of smoking status at diagnosis was highly associated with that of after diagnosis. Only 6 past smokers at diagnosis (0.9%) commenced smoking after colorectal cancer diagnosis. Among current smokers at colorectal cancer diagnosis, 58.7% (71 of 121) of them had continued smoking after diagnosis. Current smokers were associated with female sex, younger age, and earlier year of diagnosis. During the median follow-up time of 11.8 years (interquartile range = 7.2-16.4 years) for all censored patients, there were 879 all-cause deaths, including 428 colorectal cancer–specific deaths.

**Table 1.** Clinical, pathological, and molecular characteristics of colorectal cancer patients according to smoking status at diagnosis

		Smoking status at diagnosis			
	Patients	Never	Past	Current	*P* ^b^
Characteristics^a^	(n = 1474)	(n = 601)	(n = 727)	(n = 146)	
Sex, No. (%)					<.001
Female: NHS	844 (57.3)	351 (58.4)	389 (53.5)	104 (71.2)	
Male: HPFS	630 (42.7)	250 (41.6)	338 (46.5)	42 (28.8)	
Mean age ± SD, y	69.2 ± 9.1	68.8 ± 9.6	70.2 ± 8.6	65.6 ± 7.8	<.001
Year of diagnosis, No. (%)					<.001
1995 or before	514 (34.9)	209 (34.8)	228 (31.4)	77 (52.7)	
1996-2000	430 (29.2)	173 (28.8)	215 (29.6)	42 (28.8)	
2001-2014	530 (36.0)	219 (36.4)	284 (39.0)	27 (18.5)	
Family history of colorectal cancer in first-degree relative(s), No. (%)					<.79
Absent	1185 (80.6)	487 (81.4)	582 (80.2)	116 (79.5)	
Present	285 (19.4)	111 (18.6)	144 (19.8)	30 (20.5)	
BMI at diagnosis, No. (%)					.18
<25 kg/m^2^	589 (40.0)	249 (41.4)	278 (38.3)	62 (42.5)	
25 to <30 kg/m^2^	607 (41.2)	234 (38.9)	307 (42.3)	66 (45.2)	
≥30 kg/m^2^	277 (18.8)	118 (19.6)	141 (19.4)	18 (12.3)	
Alcohol consumption at diagnosis, No. (%)					<.001
0 g/d	498 (33.9)	262 (43.6)	185 (25.6)	51 (34.9)	
0 to <15 g/d	772 (52.6)	293 (48.8)	408 (56.5)	71 (48.6)	
≥15 g/d	199 (13.6)	46 (7.7)	129 (17.9)	24 (16.4)	
Physical activity at diagnosis (METS-h/wk)^c^, No. (%)					.06
Lowest	498 (35.6)	203 (35.9)	236 (33.4)	59 (45.7)	
Second	328 (23.4)	120 (21.2)	173 (24.5)	35 (27.1)	
Third	256 (18.3)	109 (19.3)	125 (17.7)	22 (17.1)	
Highest	318 (22.7)	133 (23.5)	172 (24.4)	13 (10.1)	
Processed meat intake at diagnosis, serving/d	0.15 ± 0.16	0.14 ± 0.16	0.15 ± 0.16	0.20 ± 0.17	<.001
Total fiber intake at diagnosis, g/d	21.0 ± 6.9	22.0 ± 7.5	20.9 ± 6.4	17.2 ± 5.0	<.001
Smoking status after diagnosis, No. (%)					<.001
Noncurrent smoker	1211 (94.0)	503 (100.0)	658 (99.1)	50 (41.3)	
Current smoker	77 (6.0)	0 (0.0)	6 (0.9)	71 (58.7)	
Tumor location, No. (%)					.55
Proximal colon	718 (48.9)	296 (49.3)	357 (49.3)	65 (44.8)	
Distal colon	440 (30.0)	185 (30.8)	213 (29.4)	42 (29.0)	
Rectum	311 (21.2)	119 (19.8)	154 (21.3)	38 (26.2)	
AJCC disease stage, No. (%)					.04
I	351 (26.1)	151 (27.6)	181 (27.3)	19 (14.1)	
II	429 (31.9)	167 (30.5)	213 (32.2)	49 (36.3)	
III	378 (28.1)	157 (28.7)	180 (27.2)	41 (30.4)	
IV	186 (13.8)	72 (13.2)	88 (13.3)	26 (19.3)	
Tumor differentiation, No. (%)					.51
Well to moderate	1313 (89.8)	541 (90.6)	647 (89.6)	125 (87.4)	
Poor	149 (10.2)	56 (9.4)	75 (10.4)	18 (12.6)	
MSI status, No. (%)					.16
Non–MSI-high	1074 (83.5)	438 (85.9)	528 (82.0)	108 (81.2)	
MSI-high	213 (16.6)	72 (14.1)	116 (18.0)	25 (18.8)	
CIMP status, No. (%)					.32
CIMP-low or negative	1019 (81.7)	409 (83.5)	506 (81.1)	104 (78.2)	
CIMP-high	228 (18.3)	81 (16.5)	118 (18.9)	29 (21.8)	
Mean LINE-1 methylation level ± SD	63.6 ± 10.0	63.5 ± 10.2	63.6 ± 10.0	63.4 ± 9.7	.98
KRAS mutation, No. (%)					.23
Wild type	720 (58.6)	291 (59.2)	344 (56.9)	85 (64.9)	
Mutant	508 (41.4)	201 (40.9)	261 (43.1)	46 (35.1)	
BRAF mutation, No. (%)					.73
Wild type	1100 (84.6)	442 (85.5)	544 (83.8)	114 (85.1)	
Mutant	200 (15.4)	75 (14.5)	105 (16.2)	20 (14.9)	
PIK3CA mutation, No. (%)					.53
Wild type	1013 (83.7)	400 (82.3)	512 (84.5)	101 (85.6)	
Mutant	197 (16.3)	86 (17.7)	94 (15.5)	17 (14.4)	
Tumor-infiltrating lymphocytes, No. (%)					.45
Negative or low	1,100 (74.8)	458 (76.3)	538 (74.2)	104 (71.7)	
Intermediate or high	370 (25.2)	142 (23.7)	187 (25.8)	41 (28.3)	
Intratumoral periglandular reaction, No. (%)					.77
Negative or low	191 (13.0)	80 (13.4)	90 (12.4)	21 (14.4)	
Intermediate or high	1,279 (87.0)	519 (86.6)	635 (87.6)	125 (85.6)	
Peritumoral lymphocytic reaction, No. (%)					.32
Negative or low	209 (14.3)	92 (15.4)	93 (12.9)	24 (16.4)	
Intermediate or high	1,256 (85.7)	506 (84.6)	628 (87.1)	122 (83.6)	
Crohn’s-like lymphoid reaction, No. (%)					.02
Negative or low	915 (75.8)	391 (80.0)	440 (73.0)	84 (73.0)	
Intermediate or high	292 (24.2)	98 (20.0)	163 (27.0)	31 (27.0)	

a Percentage (%) indicates the proportion of patients with a specific clinical, pathological, or molecular characteristic in patients or in strata of smoking status at diagnosis. AJCC = American Joint Committee on Cancer; BMI = body mass index; CIMP = CpG island methylator phenotype; HPFS = Health Professionals Follow-up Study; LINE-1 = long interspersed nucleotide element-1; METS = metabolic equivalent task score; MSI = microsatellite instability; NHS = Nurses’ Health Study.

b To compare characteristics between subgroups, we used the χ^2^ test for categorical variables and an analysis of variance for continuous variables. To compare continuous variables, an analysis of variance was performed.

c Physical activity was categorized into 4 categories (female: 0 to <5, 5-11.5, 11.5 to <22, and ≥22 METS-h/wk; male: 0 to <10, 10-22.5, 22.5 to <41.5, and ≥41.5 METS-h/wk).

**Figure 1.**
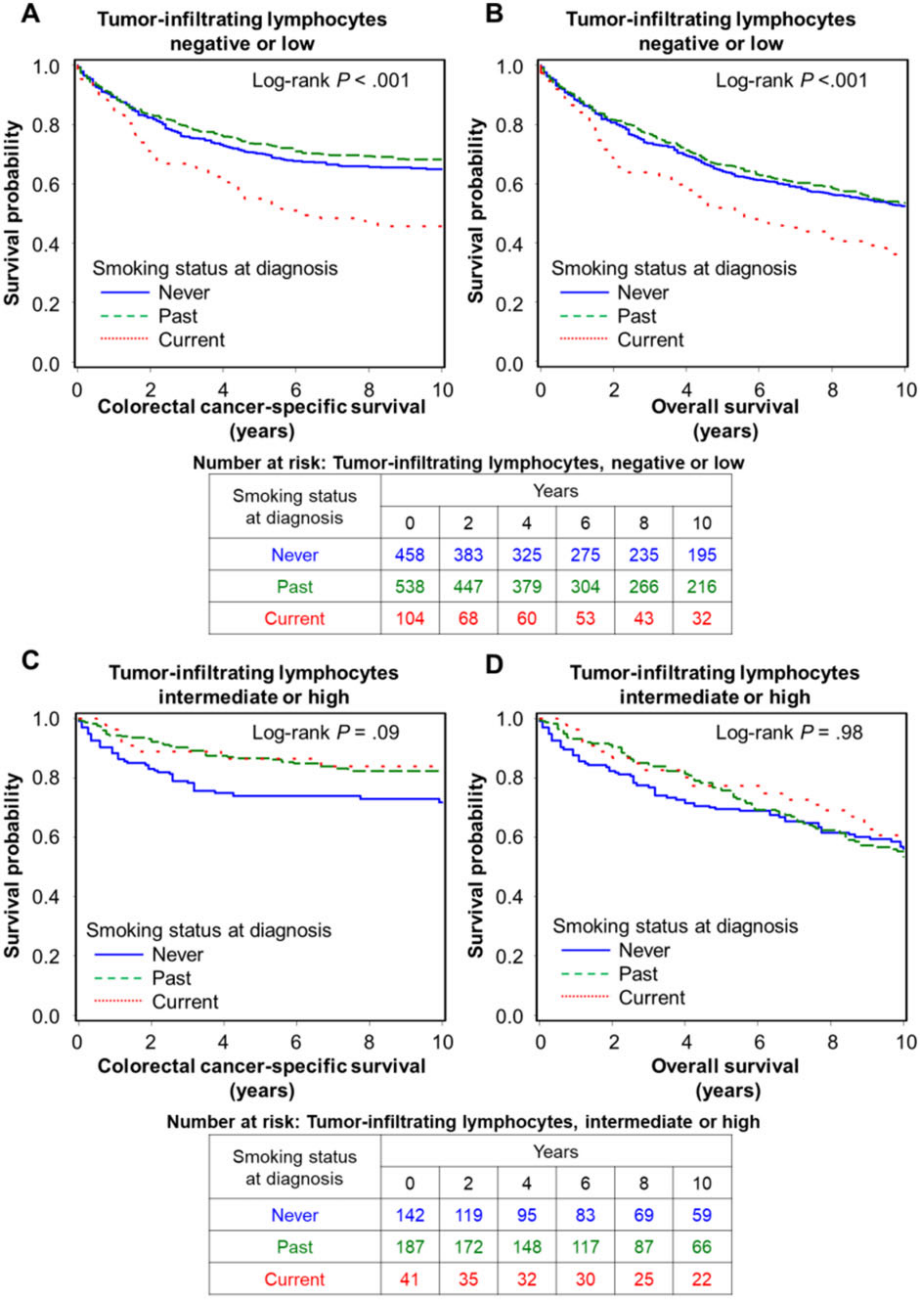
Inverse probability weighting–adjusted Kaplan-Meier curves of colorectal cancer–specific and overall survival according to smoking status at diagnosis in strata of tumor-infiltrating lymphocytes. The *P* values were calculated using the weighted 2-sided log-rank test (2-sided). **A** and **B**) Colorectal cancer with negative or low tumor-infiltrating lymphocytes. **C** and **D**) Colorectal cancer with intermediate or high tumor-infiltrating lymphocytes.

In our primary hypothesis testing, we evaluated the association of smoking status at diagnosis with colorectal cancer–specific survival according to tumor lymphocytic reaction. We found a trend of a statistical interaction between smoking status at diagnosis and TIL in relation to colorectal cancer–specific survival in the IPW-adjusted Cox model (*P*_interaction_ = .009; Table 2). Supplementary Table 1 (available online) shows the final model of the IPW-adjusted multivariable Cox regression model. Similar findings were observed when we treated smoking status as binary variables (noncurrent [never and past] vs current; never vs ever [past and current]) (Supplementary Table 2 available online). Compared with never smokers, current smokers were associated with higher colorectal cancer–specific mortality in tumors with negative or low TIL (multivariable-adjusted HR = 1.50, 95% CI = 1.10 to 2.06) but not in tumors with intermediate or high TIL (multivariable-adjusted HR = 0.43, 95% CI = 0.16 to 1.12). Figure 1 shows IPW-adjusted Kaplan-Meier survival curves for colorectal cancer–specific and overall survival according to smoking status at diagnosis in strata of TIL grade. We observed no statistically significant interaction of smoking status at diagnosis with intratumoral periglandular reaction, peritumoral lymphocytic reaction, and Crohn’s-like lymphoid reaction, respectively. Similar results were obtained by Cox regression analyses without the IPW (Supplementary Table 3 available online). We also performed analyses stratified by sex (ie, cohort) in Supplementary Table 4 (available online) and by colon and rectum in Supplementary Table 5 (available online). The results showed that, compared with never smokers, current smokers were consistently associated with higher colorectal cancer–specific mortality in patients with negative or low TIL in each stratum of women (NHS), men (HPFS), colon cancer, and rectal cancer.

**Table 2.** Smoking status at diagnosis and colorectal cancer mortality in strata of levels of lymphocytic reaction patterns

Characteristics		Colorectal cancer–specific mortality^a^^,b^			Overall mortality^a^^,b^		
	No. of cases	No. of events	Univariate HR (95% CI)	Multivariable HR (95% CI)^c^	No. of events	Univariate HR (95% CI)	Multivariable HR (95% CI)^c^
TIL (n = 1470)							
Negative or low							
Never smoker	458	141	1.00 (referent)	1.00 (referent)	262	1.00 (referent)	1.00 (referent)
Past smoker	538	158	0.89 (0.70 to 1.13)	0.80 (0.63 to 1.02)	320	1.05 (0.87 to 1.27)	0.95 (0.79 to 1.14)
Current smoker	104	54	1.79 (1.30 to 2.45)	1.50 (1.10 to 2.06)	83	1.64 (1.24 to 2.15)	1.93 (1.47 to 2.55)
Intermediate or high							
Never smoker	142	36	1.00 (referent)	1.00 (referent)	80	1.00 (referent)	1.00 (referent)
Past smoker	187	30	0.53 (0.32 to 0.91)	0.54 (0.31 to 0.94)	101	0.95 (0.71 to 1.28)	0.85 (0.62 to 1.15)
Current smoker	41	7	0.47 (0.20 to 1.13)	0.43 (0.16 to 1.12)	31	0.92 (0.60 to 1.41)	1.09 (0.68 to 1.75)
*P*_interaction_^d^			.003	.009		.06	.09
*P*_interaction_ (noncurrent vs current)^e^			.02	.03		.03	.04
Intratumoral periglandular reaction (n = 1470)							
Negative or low							
Never smoker	80	41	1.00 (referent)	1.00 (referent)	54	1.00 (referent)	1.00 (referent)
Past smoker	90	34	0.64 (0.40 to 1.03)	0.67 (0.42 to 1.07)	51	1.11 (0.68 to 1.81)	0.85 (0.56 to 1.28)
Current smoker	21	10	1.02 (0.48 to 2.18)	0.89 (0.43 to 1.82)	14	1.41 (0.67 to 2.95)	1.29 (0.60 to 2.78)
Intermediate or high							
Never smoker	519	136	1.00 (referent)	1.00 (referent)	288	1.00 (referent)	1.00 (referent)
Past smoker	635	153	0.86 (0.67 to 1.10)	0.78 (0.60 to 1.00)	369	1.03 (0.87 to 1.22)	0.92 (0.78 to 1.09)
Current smoker	125	52	1.54 (1.11 to 2.15)	1.28 (0.90 to 1.81)	101	1.41 (1.11 to 1.80)	1.68 (1.30 to 2.17)
*P*_interaction_^d^			.24	.33		.92	.59
*P*_interaction_ (noncurrent vs current)^e^			.46	.41		.93	.71
Peritumoral lymphocytic reaction (n = 1465)							
Negative or low							
Never smoker	92	48	1.00 (referent)	1.00 (referent)	66	1.00 (referent)	1.00 (referent)
Past smoker	93	43	0.79 (0.51 to 1.22)	0.99 (0.63 to 1.56)	58	1.08 (0.65 to 1.78)	0.93 (0.62 to 1.38)
Current smoker	24	13	0.97 (0.49 to 1.94)	0.86 (0.46 to 1.61)	19	1.24 (0.67 to 2.29)	1.38 (0.76 to 2.51)
Intermediate or high							
Never smoker	506	129	1.00 (referent)	1.00 (referent)	276	1.00 (referent)	1.00 (referent)
Past smoker	628	144	0.83 (0.65 to 1.07)	0.73 (0.56 to 0.94)	361	1.04 (0.87 to 1.23)	0.92 (0.77 to 1.09)
Current smoker	122	49	1.54 (1.10 to 2.16)	1.29 (0.90 to 1.84)	96	1.43 (1.11 to 1.84)	1.65 (1.26 to 2.16)
*P*_interaction_^d^			.35	.65		.83	.74
*P*_interaction_ (noncurrent vs current)^e^			.22	.16		.61	.61
Crohn’s-like lymphoid reaction (n = 1207)							
Negative or low							
Never smoker	391	131	1.00 (referent)	1.00 (referent)	235	1.00 (referent)	1.00 (referent)
Past smoker	440	129	0.81 (0.63 to 1.05)	0.81 (0.62 to 1.05)	260	0.97 (0.78 to 1.19)	0.94 (0.77 to 1.15)
Current smoker	84	43	1.63 (1.16 to 2.29)	1.37 (0.96 to 1.96)	67	1.45 (1.04 to 2.01)	1.78 (1.28 to 2.48)
Intermediate or high							
Never smoker	98	20	1.00 (referent)	1.00 (referent)	53	1.00 (referent)	1.00 (referent)
Past smoker	163	21	0.46 (0.24 to 0.88)	0.42 (0.21 to 0.83)	83	0.91 (0.66 to 1.27)	0.72 (0.51 to 1.00)
Current smoker	31	5	0.55 (0.19 to 1.61)	0.65 (0.23 to 1.88)	23	1.08 (0.72 to 1.60)	1.30 (0.84 to 2.02)
*P*_interaction_^d^			.05	.09		.43	.41
*P*_interaction_ (noncurrent vs current)^e^			.16	.53		.26	.61

^a^ IPW was applied to reduce a bias due to the availability of tumor tissue after cancer diagnosis (see “Statistical Analysis” subsection for details). AJCC = American Joint Committee on Cancer; CI = confidence interval; HR = hazard ratio; IPW = inverse probability weighting; TIL = tumor-infiltrating lymphocytes.

^b^ Hazard ratios were estimated for each stratum on the basis of the patients with smoking status at diagnosis, using a reparameterization of the interaction term in a single regression model for the stratified analyses.

^c^ The multivariable Cox regression model initially included age, sex, year of diagnosis, family history of colorectal cancer, body mass index at diagnosis, alcohol consumption at diagnosis, physical activity at diagnosis, processed meat intake at diagnosis, total fiber intake at diagnosis, tumor location, tumor differentiation, AJCC disease stage, microsatellite instability, CpG island methylator phenotype, long interspersed nucleotide element-1 methylation level, *KRAS* mutation, *BRAF* mutation, and *PIK3CA* mutation. A backward elimination with a threshold of *P *=* *.05 was used to select variables in the final models.

^d^ *P*_interaction_ was calculated using the Wald test for the cross-product of smoking status at diagnosis (ordinal; never, past, and current) and lymphocytic reaction status (binary; negative or low and intermediate or high) in Cox regression model.

^e^ *P*_interaction_ was calculated using the Wald test for the cross-product of smoking status at diagnosis (binary; noncurrent [never and past] vs current) and lymphocytic reaction status (binary; negative/low and intermediate/high) in Cox regression model.

In a secondary analysis, we evaluated the survival interaction between cumulative pack-years at diagnosis and lymphocytic reaction. We found a consistent interaction between cumulative pack-years at diagnosis and TIL in relation to colorectal cancer–specific survival (*P*_interaction_ = .02; Table 3). Additionally, past smokers, including those who had quit within the past 10 years, did not show this interaction with survival as observed with current smoking and survival (*P*_interaction_ = .01; Supplementary Table 6 available online). Although statistical power was limited, compared with never smokers, current smokers after diagnosis might be associated with higher colorectal cancer–specific mortality in patients with negative or low TIL, adjusting for prediagnostic cumulative pack-years (Supplementary Table 7 available online).

**Table 3.** Pack-years of cigarettes at diagnosis and colorectal cancer mortality in strata of levels of lymphocytic reaction patterns

Characteristics		Colorectal cancer–specific mortality^a^^,b^			Overall mortality^a^^,b^		
	No. of cases	No. of events	Univariate HR (95% CI)	Multivariable HR (95% CI)^c^	No. of events	Univariate HR (95% CI)	Multivariable HR (95% CI)^c^
TIL (n = 1430)							
Negative or low							
Pack-years = 0	458	141	1.00 (referent)	1.00 (referent)	262	1.00 (referent)	1.00 (referent)
Pack-years = 1-19	260	71	0.81 (0.60 to 1.10)	0.75 (0.54 to 1.03)	135	0.87 (0.68 to 1.10)	0.81 (0.64 to 1.03)
Pack-years = 20-39	180	61	1.07 (0.78 to 1.47)	0.98 (0.73 to 1.31)	116	1.18 (0.91 to 1.52)	1.22 (0.96 to 1.54)
Pack-years ≥40	169	68	1.40 (1.04 to 1.88)	1.17 (0.86 to 1.58)	130	1.66 (1.31 to 2.09)	1.47 (1.16 to 1.86)
Intermediate or high							
Pack-years = 0	142	36	1.00 (referent)	1.00 (referent)	80	1.00 (referent)	1.00 (referent)
Pack-years = 1-19	81	11	0.51 (0.24 to 1.08)	0.49 (0.22 to 1.08)	36	0.81 (0.53 to 1.23)	0.79 (0.52 to 1.21)
Pack-years = 20-39	69	12	0.50 (0.25 to 1.02)	0.60 (0.28 to 1.29)	40	0.88 (0.61 to 1.27)	0.85 (0.59 to 1.22)
Pack-years ≥40	71	13	0.57 (0.29 to 1.11)	0.49 (0.24 to 1.01)	54	1.23 (0.88 to 1.71)	1.10 (0.76 to 1.60)
*P*_interaction_^d^			.006	.02		.08	.07
Intratumoral periglandular reaction (n = 1430)							
Negative or low							
Pack-years = 0	80	41	1.00 (referent)	1.00 (referent)	54	1.00 (referent)	1.00 (referent)
Pack-years = 1-19	35	13	0.58 (0.29 to 1.14)	0.66 (0.34 to 1.31)	18	0.92 (0.48 to 1.76)	0.74 (0.41 to 1.34)
Pack-years = 20-39	34	17	1.01 (0.57 to 1.77)	0.91 (0.52 to 1.60)	24	1.76 (1.04 to 2.97)	1.41 (0.87 to 2.26)
Pack-years ≥40	35	11	0.60 (0.29 to 1.23)	0.61 (0.29 to 1.28)	19	0.98 (0.50 to 1.92)	0.78 (0.40 to 1.50)
Intermediate or high							
Pack-years = 0	519	136	1.00 (referent)	1.00 (referent)	288	1.00 (referent)	1.00 (referent)
Pack-years = 1-19	306	68	0.80 (0.59 to 1.10)	0.70 (0.50 to 0.98)	152	0.86 (0.69 to 1.06)	0.79 (0.63 to 0.99)
Pack-years = 20-39	215	56	0.92 (0.66 to 1.28)	0.87 (0.63 to 1.20)	132	1.04 (0.83 to 1.30)	1.05 (0.85 to 1.30)
Pack-years ≥40	206	71	1.34 (1.00 to 1.80)	1.13 (0.82 to 1.55)	166	1.63 (1.33 to 2.00)	1.45 (1.16 to 1.80)
P_interaction_^d^			.13	.22		.54	.33
Peritumoral lymphocytic reaction (n = 1425)							
Negative or low							
Pack-years = 0	92	48	1.00 (referent)	1.00 (referent)	66	1.00 (referent)	1.00 (referent)
Pack-years = 1-19	40	20	0.87 (0.49 to 1.54)	1.11 (0.61 to 2.05)	27	1.32 (0.75 to 2.34)	1.04 (0.64 to 1.68)
Pack-years = 20-39	32	15	0.88 (0.48 to 1.59)	1.08 (0.59 to 1.99)	21	1.25 (0.71 to 2.20)	1.36 (0.83 to 2.22)
Pack-years ≥40	38	18	0.77 (0.42 to 1.41)	0.76 (0.42 to 1.35)	25	0.91 (0.48 to 1.74)	0.84 (0.48 to 1.47)
Intermediate or high							
Pack-years = 0	506	129	1.00 (referent)	1.00 (referent)	276	1.00 (referent)	1.00 (referent)
Pack-years = 1-19	298	61	0.75 (0.54 to 1.04)	0.64 (0.46 to 0.91)	142	0.82 (0.66 to 1.02)	0.75 (0.60 to 0.95)
Pack-years = 20-39	216	58	0.96 (0.69 to 1.34)	0.87 (0.63 to 1.20)	135	1.09 (0.87 to 1.37)	1.07 (0.87 to 1.33)
Pack-years ≥40	203	64	1.28 (0.94 to 1.74)	1.08 (0.78 to 1.50)	160	1.68 (1.38 to 2.04)	1.45 (1.16 to 1.81)
*P*_interaction_^d^			.20	.45		.11	.13
Crohn’s-like lymphoid reaction (n = 1174)							
Negative/low							
Pack-years = 0	391	131	1.00 (referent)	1.00 (referent)	235	1.00 (referent)	1.00 (referent)
Pack-years = 1-19	215	61	0.77 (0.55 to 1.06)	0.73 (0.52 to 1.04)	108	0.77 (0.59 to 1.01)	0.79 (0.61 to 1.04)
Pack-years = 20-39	135	46	1.03 (0.73 to 1.46)	1.06 (0.77 to 1.45)	88	1.14 (0.85 to 1.52)	1.25 (0.97 to 1.60)
Pack-years ≥40	148	54	1.14 (0.83 to 1.58)	1.01 (0.72 to 1.42)	113	1.44 (1.10 to 1.87)	1.33 (1.01 to 1.75)
Intermediate/high							
Pack-years = 0	98	20	1.00 (referent)	1.00 (referent)	53	1.00 (referent)	1.00 (referent)
Pack-years = 1-19	70	6	0.33 (0.12 to 0.90)	0.29 (0.10 to 0.84)	30	0.75 (0.50 to 1.13)	0.61 (0.40 to 0.94)
Pack-years = 20-39	58	9	0.58 (0.25 to 1.35)	0.66 (0.28 to 1.55)	31	0.91 (0.60 to 1.36)	0.90 (0.60 to 1.35)
Pack-years ≥40	59	11	0.60 (0.28 to 1.31)	0.54 (0.25 to 1.18)	43	1.32 (0.93 to 1.86)	1.13 (0.78 to 1.64)
P_interaction_^d^			.13	.15		.56	.69

^a^ IPW was applied to reduce a bias due to the availability of tumor tissue after cancer diagnosis (see “Statistical Analysis” subsection for details). AJCC = American Joint Committee on Cancer; CI = confidence interval; HR = hazard ratio; IPW = inverse probability weighting; TIL = tumor-infiltrating lymphocytes.

^b^ Hazard ratios were estimated for each stratum on the basis of the patients with pack-years of cigarettes at diagnosis, using a reparameterization of the interaction term in a single regression model for the stratified analyses.

^c^ The multivariable Cox regression model initially included age, sex, year of diagnosis, family history of colorectal cancer, body mass index at diagnosis, alcohol consumption at diagnosis, physical activity at diagnosis, processed meat intake at diagnosis, total fiber intake at diagnosis, tumor location, tumor differentiation, AJCC disease stage, microsatellite instability, CpG island methylator phenotype, long interspersed nucleotide element-1 methylation level, *KRAS* mutation, *BRAF* mutation, and *PIK3CA* mutation. A backward elimination with a threshold of *P *=* *.05 was used to select variables in the final models.

^d^ *P*_interaction_ was calculated using the Wald test for the cross-product of pack-years of cigarettes at diagnosis (ordinal; 0, 1-19, 20-39, and ≥40) and lymphocytic reaction status (binary; negative or low and intermediate or high) in Cox regression model.

We constructed prediction models for 5-year colorectal cancer–specific and overall survival based on smoking status at diagnosis, TIL status, and other clinical prognostic variables (Supplementary Methods; Supplementary Table 8 available online).

## Discussion

We conducted this study to test the hypothesis that the association of smoking status at diagnosis with mortality might differ by lymphocytic reaction patterns in tumor tissue. Utilizing 1474 colorectal cancer patients with detailed information on host lifestyle risk factors as well as tumor pathological and molecular data among 4420 incident colorectal cancer patients in the 2 US prospective cohort studies, we found that current smokers had worse colorectal cancer–specific survival in the negative or low TIL group but not in the intermediate or high TIL group. Similar survival interactions were obtained between TIL grade and pack-years at diagnosis. Although validation in independent datasets is warranted, our findings provided population-based evidence that current smoking influences colorectal cancer mortality via modified effects by host immunity.

Cigarette smoke is a complex mixture of more than 4500 chemicals, many of which have been shown to modulate the function of immune cells (7,32), suggesting that these chemicals can result in the suppression of antitumor immunity, including both innate and adaptive immune response. These chemicals can enter the local tissue microenvironment and constitute etiologic field effects (33–36). Nicotine, which is a major component of cigarette smoke, and its metabolite cotinine can facilitate tumor progression by impairment of the immune system. CHRN (cholinergic receptors nicotinic subunits, also known as nicotinic acetylcholine receptors)-mediated signaling, for example, activates several tumor-promoting networks such as RAS (RAS type GTPase family)-RAF (RAF kinase)-MAPK (mitogen-activated protein kinases) and JAK (jak family tyrosine kinases)-STAT3 (signal transducer and activator of transcription 3) pathways (32,37,38). An experimental study showed that nicotine can increase immune suppression mediated by regulatory T cells via cholinergic receptor nicotinic alpha 7 subunit (39). In addition, immunosuppression effects in nicotine-treated rats were sustained for several weeks after cessation of nicotine administration, indicating that the immunosuppression effects of nicotine can have sustained effects after initial nicotine exposure (40). Acrolein, which is a toxic unsaturated aldehyde constituent of cigarette smoke, could suppress antitumor immunity through altering neutrophil function, resulting in decreasing responsiveness of CD8^+^ T cells to T-cell receptor triggering (41,42). Other chemicals, such as benzo (α) pyrene and hydroquinone, can also inhibit T-cell immune response (43). A population-based study showed that the number and activity of natural killer cells were decreased in regular smokers compared with nonsmokers (44). The function of the dendritic cells was suppressed in a dose- and time-dependent manner by cigarette smoke (45,46). Thus, a better understanding of the effect of smoking on antitumor immunity could have considerable clinical implications. Our findings suggest that colorectal cancer subtypes with a high-level lymphocytic reaction may be less sensitive to the adverse prognostic effects of cigarette smoking, whereas tumors with low-level lymphocytic reaction can be immunologically incompetent and may be more vulnerable to the deleterious effects of smoking.

Moreover, the immunosuppressive effect of smoking may synergize with inferior survival for colorectal cancers with weaker lymphocytic reactions. Recent studies suggest that high-level antitumor immune response may potentially contribute to better response to not only immunotherapy but also chemotherapy (12,47). Chemotherapy and radiotherapy have been shown to induce more tumor cell death in tumors containing higher numbers of immune cells than in tumors containing fewer immune cells (47). Studies also showed that current smoking was associated with shorter disease-free survival among colon cancer patients who were receiving fluorouracil-based adjuvant chemotherapy (48,49), suggesting that smoking might cause treatment resistance (50,51). Given our data suggesting the interactive influences of smoking and TIL on tumor progression, it is of interest to examine in future studies whether smoking and TIL may jointly modify responsiveness to various forms of therapy. Accordingly, integrated analyses of tumor and host characteristics, including immune profile and lifestyle factors such as smoking behavior, have become important to identify individuals with potentially better response to therapeutic and lifestyle interventions (10). Our data strengthen the link between smoking and colorectal cancer mortality, demonstrating survival interactions according to tumor immune profile and enhancing our understanding of the mechanisms through which smoking may exert its neoplastic effects via the immune system. Our data support that smoking at diagnosis may influence host antitumor responses, especially the lymphocytic reaction, influencing colorectal cancer mortality. In addition, our findings can have clinical implications. The potential health benefits of smoking cessation are underscored for cancer prevention and interception, although our findings suggest that patients with more than 10 years of smoking cessation may not have strong survival benefits relative to continuing smoking within the negative or low TIL subgroup compared with patients with less than 10 years of smoking cessation. Particularly, individuals with tumors presenting with negative or low TIL may represent a patient population in need of additional measures to improve survival, including advocacy for immediate smoking cessation or the identification of more aggressive treatment strategies compared with never or past smokers with limited pack-years of exposure.

Our study has several limitations. First, there was the possibility of unmeasured confounding. Second, data on cancer treatment were limited. However, distribution of treatment including the use of chemotherapy and molecular targeting therapy and its regimen unlikely substantially differed by lymphocytic reaction pattern in cancer tissue, because these data were not available at the time of cancer treatment decisions. Third, data on cancer recurrence were not available in this study. Given that median cancer recurrence (metastasis) was approximately 10 to 20 months (52), colorectal cancer–specific survival can be a reasonable clinical outcome for colorectal cancer in a population-based study with long-term follow-up. Fourth, given that current smoking at diagnosis was associated with smoking after diagnosis, we could not evaluate whether quitting after diagnosis would confer the same benefit as not smoking at the time of diagnosis. Lastly, the availability of tissue after colorectal cancer diagnosis might have introduced bias. However, IPW methods were used in all survival analyses to specifically reduce this potential bias.

The major strength of this study was the use of a molecular pathological epidemiology (8–10) database of colorectal carcinoma patients in 2 US nationwide prospective cohort studies that integrate clinicopathological and molecular features, long-term survival data, and lifestyle factors, including smoking status and tumor immune profile. The concept of molecular pathological epidemiology has been used widely (53–58). In particular, examining the interactions between environmental exposures and immune cells in the tumor microenvironment is an important research direction (59) and may inform strategies to improve clinical outcomes. This comprehensive database allowed us to examine an interactive prognostic association of smoking status at diagnosis and tumor lymphocytic reactions and control for a variety of potential confounders and selection bias due to tumor tissue availability. Our study population was derived from a large number of colorectal cancer patients from hospitals throughout the United States, which can contribute to increased generalizability of our findings. Nevertheless, our findings should be validated in independent studies.

In conclusion, the association of smoking behavior at diagnosis with colorectal cancer survival appears to differ by the host immune system, and tumors with negative or low lymphocytic reaction resulted in poor survival among current smokers compared with never smokers. Our findings emphasize the link between smoking and tumor immunity, both of which may interactively influence colorectal cancer progression.

## Funding

This work was supported by US National Institutes of Health (NIH) grants (P01 CA87969; UM1 CA186107; P01 CA55075; UM1 CA167552; U01 CA167552; P50 CA127003 to C.S.F.; R01 CA118553 to C.S.F.; R01 CA169141 to C.S.F.; R01 CA137178 to A.T.C.; K24 DK098311 to A.T.C.; R35 CA197735 to S.O.; R01 CA151993 to S.O.; R01 CA248857 to S.O.; K07 CA190673 to R.N.; K07 CA188126 to X.Z., and R01 CA225655 to J.K.L.); by Cancer Research UK’s Grand Challenge Award (OPTIMISTICC; UK C10674/A27140 to M.G. and S.O.); by Nodal Award (2016-20) from the Dana-Farber Harvard Cancer Center (to S.O.); by Stand Up to Cancer Colorectal Cancer Dream Team Translational Research Grant (SU2C-AACR-DT22-17 to C.S.F. and M.G.), administered by the American Association for Cancer Research, a scientific partner of SU2C; and by grants from the Project P Fund, The Friends of the Dana-Farber Cancer Institute, Bennett Family Fund, and the Entertainment Industry Foundation through National Colorectal Cancer Research Alliance and SU2C. K.F. and K.H. were supported by fellowship grants from the Uehara Memorial Foundation. K.F. was supported by fellowship grants from the Grant of The Clinical Research Promotion Foundation (2018). Y.C. and L.L. were supported by a scholarship grant from Chinese Scholarship Council. K.H. was supported by fellowship grants from the Mitsukoshi Health and Welfare Foundation. T.U. and K.A. were supported by a grant from Overseas Research Fellowship (201960541 to T.U.; 201860083 to K.A) from Japan Society for the Promotion of Science. A.T.C. is a Stuart and Suzanne Steele MGH Research Scholar. M.G. is supported by an ASCO Conquer Cancer Foundation Career Development Award.

## Notes

**Role of the funders:** The funders had no role in study design, data collection and analysis, decision to publish, or preparation of the manuscript.

**Disclaimer:** The content is solely the responsibility of the authors and does not necessarily represent the official views of NIH.

**Disclosures:** A.T.C. previously served as a consultant for Bayer Healthcare and Pfizer Inc. This study was not funded by Bayer Healthcare or Pfizer Inc. J.A.M has received institutional research funding from Boston Biomedical, has served as an advisor/consultant to Ignyta and COTA Healthcare, and served on a grant review panel for the National Comprehensive Cancer Network funded by Taiho Pharmaceutical. C.S.F. previously served as a consultant for Agios, Bain Capital, Bayer, Celgene, Dicerna, Five Prime Therapeutics, Gilead Sciences, Eli Lilly, Entrinsic Health, Genentech, KEW, Merck, Merrimack Pharmaceuticals, Pfizer Inc, Sanofi, Taiho, and Unum Therapeutics; C.S.F. also serves as a Director for CytomX Therapeutics and owns unexercised stock options for CytomX and Entrinsic Health. R.N. is currently employed by Pfizer Inc; she contributed to this study before she became an employee of Pfizer Inc. M.G. receives research funding from Bristol-Myers Squibb and Merck. This study was not funded by any of these commercial entities. No other conflicts of interest exist. The other authors declare that they have no conflicts of interest.

**Author contributions:** All authors contributed to review and revision. M.G., J.A.N., and S.O.: developed the main concept and designed the study. R.N., A.T.C., C.S.F., M.G., and S.O.: wrote grant applications. K.F., Y.C., K.H., T.U., J.K., T.H., L.L., J.B., D.A.D, M.S., A.T.C., E.L.G., J.A.M., C.S.F., R.N., J.K.L., J.A.N., and S.O.: were responsible for collection of tumor tissue, and acquisition of epidemiologic, clinical and tumor tissue data, including histopathological, immunohistochemical, and immunofluorescent characteristics. K.F., Y.C., T.U., J.K., K.H., C.S.F., R.N., X.Z., K.W., and S.O.: performed data analysis and interpretation. K.F., Y.C., K.H., T.U., J.K., and S.O.: drafted the manuscript. M.Z., K.A., J.P.V., M.C.L., S.G., S.S., N.A., T.S.T., M.S., R.N., M.G., J.A.N., X.Z., K.W., and S.O.: contributed to editing and critical revision for important intellectual contents.

## Supplementary Material

pkaa040_Supplementary_Data
