# Supplementary material for: Smoking Status at Diagnosis and Colorectal Cancer Prognosis According to Tumor Lymphocytic Reaction
Source: JNCI Cancer Spectr. 2020 May 14;4(5):pkaa040. doi: 10.1093/jncics/pkaa040 (PMC7477375; doi:10.1093/jncics/pkaa040)
Supplement: pkaa040_Supplementary_Data [file pkaa040_supplementary_data.pdf]

Supplementary methods

Assessment of dietary intake

Data on dietary intake was collected in the two cohorts as reported previously (1,2). Dietary intake has been reported by participants on biennial questionnaires since 1980 (NHS) and 1986 (HPFS). Processed meat (hot dogs, bacon, deli meats) intake was classified as number of servings per day (1). Fiber intake was calculated using the Association of Official Analytical Chemists method (accepted by the U.S. Food and Drug Administration and the Food and Agriculture Organization of the World Health Organization for nutrition labeling purposes) (3). We adjusted processed meat intake for total caloric intake. Dietary intake at colorectal cancer diagnosis was derived from the latest available questionnaire before diagnosis.

Statistical analyses

All statistical analyses were performed using SAS software (version 9.4, SAS Institute, Cary, NC, USA). All *P* values were two-sided. Our primary hypothesis test was an assessment of a statistical interaction between smoking status at diagnosis (ordinal; never, past, and current) and lymphocytic reaction in tumor tissue (binary classification of negative/low and intermediate/high) in a multivariable Cox proportional hazards regression model using the Wald test on the cross-product. We used the two-sided α-level of 0.005 (5). Hazard ratio (HR) for smoking status at

diagnosis in strata of lymphocytic reaction components using a re-parameterization of the interaction term was also assessed in a single regression model (6).

Primary outcome endpoint of this study was colorectal cancer-specific mortality and secondary endpoint was overall mortality. For colorectal cancer-specific survival analyses, deaths of other causes and cases with missing data on cause of death were censored. Survival time was defined as the period from diagnosis of colorectal cancer to death or the end of follow-up, whichever came first.

In all survival analyses, we used covariate data of 4,420 incident colorectal cancer cases and the inverse probability weighting (IPW) method which is the method to reduce the bias of tissue selection by adding weights to each case with tumor tissue (6-8). Each case is weighted by the inverse of the probability of the tumor availability based on the multivariable-adjusted logistic regression model using the entire data set of colorectal cancer regardless of available tissue (6-8). First, the probability of tumor tissue as an outcome was estimated using the multivariable-adjusted logistic regression model, which initially included sex (female vs. male), age at diagnosis (continuous; a linear term, and a squared term), year of diagnosis (continuous; a linear term, and a squared term), family history of colorectal cancer (present vs. absent vs. missing), body mass index at diagnosis (< 25 kg/m^2 vs. 25 to 29.9 kg/m^2 vs. ≥ 30 kg/m^2 vs. missing), tumor location (cecum vs. ascending colon vs. transverse colon vs. descending colon vs. sigmoid colon vs. rectum vs. missing), and disease stage (I vs. II vs. III vs. IV vs. missing). A backward elimination with a threshold *P* of 0.20 was used to select variables for the final model. After the

selection procedure, the final model included sex, year of diagnosis (a linear term and a squared term), tumor location, and disease stage. Weights greater than the 95th percentile were truncated and set to the value of the 95th percentile to reduce outlier effects. We confirmed that results without weight truncation did not change substantially (data not shown). The IPW-adjusted Kaplan-Meier method was used to estimate the distribution of colorectal cancer-specific and overall survivals, and the weighted log-rank test was performed (9). Similar results were obtained by Cox regression analyses without the IPW.

IPW-adjusted multivariable Cox proportional hazards regression models were used to adjust for potential confounders and initially included followings: sex (i.e., cohort), age at diagnosis (continuous), year of diagnosis (continuous), family history of colorectal cancer (present vs. absent), body mass index (BMI) at diagnosis (≥ 30 vs. < 30 kg/m^2), alcohol consumption at diagnosis (< 15 vs. ≥ 15 g/day), physical activity at diagnosis (< 11.5 vs. ≥ 11.5 metabolic equivalent task score (METS)-hours/week in female; < 22.5 vs. ≥ 22.5 METS-hours/week in male), processed meat intake at diagnosis (< 0.09 vs. ≥ 0.09 servings/day in female; < 0.13 vs. ≥ 0.13 servings/day in male), total fiber intake at diagnosis (< 18.8 vs. ≥ 18.8 g/day in female; < 22.2 vs. ≥ 22.2 g/day in male), tumor location (proximal colon vs. distal colon vs. rectum), tumor differentiation (well to moderate vs. poor), disease stage (I-II vs. III-IV), MSI status (MSI-high vs. non-MSI-high vs. missing), CIMP (low/negative vs. high vs. missing), LINE-1 methylation level (continuous), *KRAS* mutation (mutant vs. wild-type vs. missing), *BRAF* mutation (mutant vs. wild-type vs. missing), and

PIK3CA mutation (mutant vs. wild-type vs. missing). A backward elimination with a threshold of *P* = 0.05 was performed to select variables for the final models. Cases with missing data [family history of colorectal cancer (0.3%), body mass index (0.1%), alcohol consumption (0.3%), physical activity (5.0%), processed meat intake (0.1%), total fiber intake (0.1%), tumor location (0.3%), tumor differentiation (0.8%), disease stage (8.8%)] were included in the majority category of a given categorical covariate to limit the degrees of freedom of the models. For cases with missing data [MSI status (13%), CIMP status (15%), LINE-1 methylation level (14%), *KRAS* mutation (17%), *BRAF* mutation (12%), and *PIK3CA* mutation (18%)], we categorized them as a separate indicator variable. We confirmed that exclusion of cases with missing data in any of the covariates did not substantially alter the results (data not shown). The proportionality of hazards assumption was assessed using a time varying covariate, which is an interaction term of survival time and smoking status at diagnosis. The proportionality of hazards assumption was generally satisfied for cancer specific survival ($P > 0.28$).

Furthermore, we investigated whether the prognostic prediction model can improve accuracy by adding smoking status at diagnosis and TIL status to the prediction model based on other clinical prognostic variables in the following approach. First, the candidate variables were initially included in a multivariate logistic regression model as follows; sex (i.e., cohort), age at diagnosis (continuous), year of diagnosis (continuous), family history of colorectal cancer (present vs. absent), body mass index (BMI) at diagnosis (≥ 30 vs. < 30 kg/m^2), alcohol

consumption at diagnosis (< 15 vs. ≥ 15 g/day), physical activity at diagnosis (female: 0 to 11.5 vs. ≥ 11.5 METS-hours/week; male: 0 to < 22.5 vs. ≥ 22.5 METS-hours/week), processed meat intake at diagnosis (< 0.09 vs. ≥ 0.09 servings/day in female; < 0.13 vs. ≥ 0.13 servings/day in male), total fiber intake at diagnosis (< 18.8 vs. ≥ 18.8 g/day in female; < 22.2 vs. ≥ 22.2 g/day in male), tumor location (proximal colon vs. distal colon vs. rectum), tumor differentiation (well to moderate vs. poor), disease stage (I-II vs. III-IV), MSI status (MSI-high vs. non-MSI-high vs. missing), CIMP (low/negative vs. high vs. missing), LINE-1 methylation level (continuous), *KRAS* mutation (mutant vs. wild-type vs. missing), *BRAF* mutation (mutant vs. wild-type vs. missing), and *PIK3CA* mutation (mutant vs. wild-type vs. missing). Then, a backward elimination with a threshold of $P = 0.05$ was performed to select variables for the final models. We finally included age, year of diagnosis, tumor location, tumor differentiation, disease stage, microsatellite instability, long interspersed nucleotide element-1 methylation level, *KRAS* mutation, and *BRAF* mutation in the prediction model for five-year colorectal cancer-specific survival. The final model for five-year overall survival included age, year of diagnosis, tumor differentiation, disease stage, microsatellite instability, and *BRAF* mutation. We also constructed prediction models by adding smoking status at diagnosis and/or TIL status. To compare the model discrimination performance, we drew receiver-operating characteristics (ROC) curves and compared the area under the ROC curve between these models.

References

1. Willett WC, Howe GR, Kushi LH. Adjustment for total energy intake in epidemiologic studies. Am J Clin Nutr 1997;65(4 Suppl):1220S-1228S; discussion 1229S-1231S.

2. Song M, Wu K, Meyerhardt JA*, et al.* Fiber Intake and Survival After Colorectal Cancer Diagnosis. JAMA Oncol 2018;4(1):71-79.

3. Prosky L, Asp NG, Furda I*, et al.* Determination of total dietary fiber in foods and food products: collaborative study. J Assoc Off Anal Chem 1985;68(4):677-9.

4. Pan A, Sun Q, Bernstein AM*, et al.* Red meat consumption and mortality: results from 2 prospective cohort studies. Arch Intern Med 2012;172(7):555-63.

5. Benjamin DJ, Berger JO, Johannesson M*, et al.* Redefine statistical significance. Nat Hum Behav 2018;2(1):6-10.

6. Hamada T, Cao Y, Qian ZR*, et al.* Aspirin Use and Colorectal Cancer Survival According to Tumor CD274 (Programmed Cell Death 1 Ligand 1) Expression Status. J Clin Oncol 2017;35(16):1836-1844.

7. Liu L, Nevo D, Nishihara R*, et al.* Utility of inverse probability weighting in molecular pathological epidemiology. Eur J Epidemiol 2018;33(4):381-392.

8. Seaman SR, White IR. Review of inverse probability weighting for dealing with missing data. Stat Methods Med Res 2013;22(3):278-95.

9. Xie J, Liu C. Adjusted Kaplan-Meier estimator and log-rank test with inverse probability of treatment weighting for survival data. Stat Med 2005;24(20):3089-110.

Supplementary table S1. Colorectal cancer mortality according to smoking status at diagnosis in strata of tumor-infiltrating lymphocytes (the final multivariable models)

	Colorectal cancer-specific mortality	Overall mortality
	Multivariable HR (95% CI)*,†,‡	Multivariable HR (95% CI)*,†,‡
**Tumor-infiltrating lymphocytes**		
Negative/low		
Never smoker	1 (referent)	1 (referent)
Past smoker	0.80 (0.63-1.02)	0.95 (0.79-1.14)
Current smoker	1.50 (1.10-2.06)	1.93 (1.47-2.55)
Intermediate/high		
Never smoker	1 (referent)	1 (referent)
Past smoker	0.54 (0.31-0.94)	0.85 (0.62-1.15)
Current smoker	0.43 (0.16-1.12)	1.09 (0.68-1.75)
Age (per 10-year increase)	1.29 (1.13-1.47)	1.90 (1.71-2.13)
Female	Did not remain in this model	1.25 (1.08-1.46)
Year of diagnosis (per 5-year increase)	0.86 (0.77-0.95)	Did not remain in this model
Tumor location		
Proximal colon	1 (referent)	
Distal colon	0.76 (0.59-0.97)	Did not remain in this model
Rectum	0.89 (0.68-1.17)	
AJCC disease stage		
I-II	1 (referent)	1 (referent)
III-IV	5.74 (4.51-7.32)	2.30 (2.00-2.64)
Tumor differentiation		
Well to moderate	1 (referent)	1 (referent)
Poor	2.70 (1.94-3.75)	1.76 (1.38-2.26)
MSI status		
Non-MSI-high	1 (referent)	Did not remain in this model
MSI-high	0.27 (0.15-0.48)	
LINE-1 methylation level (per 30% decrease)	1.69 (1.22-2.34)	1.42 (1.10-1.82)
BRAF status		
Wild type	1 (referent)	1 (referent)
Mutant	1.89 (1.33-2.69)	1.29 (1.00-1.65)

* IPW was applied to reduce a bias due to the availability of tumor tissue after cancer diagnosis (see "Statistical Analysis" subsection for details).

† HRs were estimated for each stratum on the basis of the cases with smoking status at diagnosis, using a re-parameterization of the interaction term in a single regression model for the stratified analyses.

‡ The multivariable Cox regression model initially included age, sex, year of diagnosis, family history of colorectal cancer, body mass index at diagnosis, alcohol consumption at diagnosis, physical activity at diagnosis, processed meat intake at diagnosis, total fiber intake at diagnosis, tumor location, tumor differentiation, AJCC disease stage, microsatellite instability, CpG island methylator phenotype, long interspersed nucleotide element-1 methylation level, *KRAS* mutation, *BRAF* mutation, and *PIK3CA* mutation. A backward elimination with a threshold of $P = 0.05$ was used to select variables in the final models.

Abbreviations: CI, confidence interval; HR, hazard ratio; IPW, inverse probability weighting; LINE-1, long interspersed nucleotide element-1; METS, metabolic equivalent task score; MSI, microsatellite instability.

Supplementary table S2. Smoking status at diagnosis and colorectal cancer mortality in strata of levels of lymphocytic reaction patterns (binary smoking status for $P_{interaction}$)

		Colorectal cancer-specific mortality*,†			Overall mortality*,†		
	No. of cases	No. of events	Univariable HR (95% CI)	Multivariable HR (95% CI)‡	No. of events	Univariable HR (95% CI)	Multivariable HR (95% CI)‡
**Tumor-infiltrating lymphocytes (n = 1,470)**							
Negative/low							
Never smoker	458	141	1 (referent)	1 (referent)	262	1 (referent)	1 (referent)
Past smoker	538	158	0.89 (0.70-1.13)	0.80 (0.63-1.02)	320	1.05 (0.87-1.27)	0.95 (0.79-1.14)
Current smoker	104	54	1.79 (1.30-2.45)	1.50 (1.10-2.06)	83	1.64 (1.24-2.15)	1.93 (1.47-2.55)
Intermediate/high							
Never smoker	142	36	1 (referent)	1 (referent)	80	1 (referent)	1 (referent)
Past smoker	187	30	0.53 (0.32-0.91)	0.54 (0.31-0.94)	101	0.95 (0.71-1.28)	0.85 (0.62-1.15)
Current smoker	41	7	0.47 (0.20-1.13)	0.43 (0.16-1.12)	31	0.92 (0.60-1.41)	1.09 (0.68-1.75)
$P_{interaction}$ (non-current vs. current)§			0.017	0.032		0.029	0.036
$P_{interaction}$ (never vs. ever)¶			0.013	0.044		0.25	0.28
**Intratumoral periglandular reaction (n = 1,470)**							
Negative/low							
Never smoker	80	41	1 (referent)	1 (referent)	54	1 (referent)	1 (referent)
Past smoker	90	34	0.64 (0.40-1.03)	0.67 (0.42-1.07)	51	1.11 (0.68-1.81)	0.85 (0.56-1.28)
Current smoker	21	10	1.02 (0.48-2.18)	0.89 (0.43-1.82)	14	1.41 (0.67-2.95)	1.29 (0.60-2.78)
Intermediate/high							
Never smoker	519	136	1 (referent)	1 (referent)	288	1 (referent)	1 (referent)
Past smoker	635	153	0.86 (0.67-1.10)	0.78 (0.60-1.00)	369	1.03 (0.87-1.22)	0.92 (0.78-1.09)
Current smoker	125	52	1.54 (1.11-2.15)	1.28 (0.90-1.81)	101	1.41 (1.11-1.80)	1.68 (1.30-2.17)
$P_{interaction}$ (non-current vs. current)§			0.46	0.41		0.93	0.71
$P_{interaction}$ (never vs. ever)¶			0.23	0.59		0.79	0.83

**Peritumoral lymphocytic reaction (n = 1,465)**							
Negative/low							
Never smoker	92	48	1 (referent)	1 (referent)	66	1 (referent)	1 (referent)
Past smoker	93	43	0.79 (0.51-1.22)	0.99 (0.63-1.56)	58	1.08 (0.65-1.78)	0.93 (0.62-1.38)
Current smoker	24	13	0.97 (0.49-1.94)	0.86 (0.46-1.61)	19	1.24 (0.67-2.29)	1.38 (0.76-2.51)
Intermediate/high							
Never smoker	506	129	1 (referent)	1 (referent)	276	1 (referent)	1 (referent)
Past smoker	628	144	0.83 (0.65-1.07)	0.73 (0.56-0.94)	361	1.04 (0.87-1.23)	0.92 (0.77-1.09)
Current smoker	122	49	1.54 (1.10-2.16)	1.29 (0.90-1.84)	96	1.43 (1.11-1.84)	1.65 (1.26-2.16)
$P_{interaction}$ (non-current vs. current)[§]			0.22	0.16		0.61	0.61
$P_{interaction}$ (never vs. ever)[¶]			0.59	0.46		0.94	0.45
**Crohn's-like lymphoid reaction (n = 1,207)**							
Negative/low							
Never smoker	391	131	1 (referent)	1 (referent)	235	1 (referent)	1 (referent)
Past smoker	440	129	0.81 (0.63-1.05)	0.81 (0.62-1.05)	260	0.97 (0.78-1.19)	0.94 (0.77-1.15)
Current smoker	84	43	1.63 (1.16-2.29)	1.37 (0.96-1.96)	67	1.45 (1.04-2.01)	1.78 (1.28-2.48)
Intermediate/high							
Never smoker	98	20	1 (referent)	1 (referent)	53	1 (referent)	1 (referent)
Past smoker	163	21	0.46 (0.24-0.88)	0.42 (0.21-0.83)	83	0.91 (0.66-1.27)	0.72 (0.51-1.00)
Current smoker	31	5	0.55 (0.19-1.61)	0.65 (0.23-1.88)	23	1.08 (0.72-1.60)	1.30 (0.84-2.02)
$P_{interaction}$ (non-current vs. current)[§]			0.16	0.53		0.26	0.61
$P_{interaction}$ (never vs. ever)[¶]			0.043	0.078		0.61	0.33

[*] IPW was applied to reduce a bias due to the availability of tumor tissue after cancer diagnosis (see "Statistical Analysis" subsection for details).

[†] HRs were estimated for each stratum on the basis of the cases with smoking status at diagnosis, using a re-parameterization of the interaction term in a single regression model for the stratified analyses.

[‡] The multivariable Cox regression model initially included age, sex, year of diagnosis, family history of colorectal cancer, body mass index at diagnosis, alcohol consumption at diagnosis, physical activity at diagnosis, processed meat intake at diagnosis, total fiber intake at diagnosis, tumor location, tumor differentiation, AJCC disease stage, microsatellite instability, CpG island methylator phenotype, long interspersed nucleotide element-1 methylation level, *KRAS* mutation, *BRAF* mutation, and *PIK3CA* mutation. A backward elimination with a threshold of $P = 0.05$ was used to select variables in the final models.

[§-¶] $P_{interaction}$ was calculated using the Wald test for the cross-product of smoking status at diagnosis [binary; non-current (never and past) and current[§]; never and ever (past and current)[¶]] and lymphocytic reaction status (binary; negative/low and intermediate/high) in Cox regression model.

Abbreviations: CI, confidence interval; HR, hazard ratio; IPW, inverse probability weighting.

Supplementary table S3. Smoking status at diagnosis and colorectal cancer mortality in strata of levels of lymphocytic reaction patterns (without inverse probability weighting method)

		Colorectal cancer-specific mortality*			Overall mortality*		
	No. of cases	No. of events	Univariable HR (95% CI)	Multivariable HR (95% CI)†	No. of events	Univariable HR (95% CI)	Multivariable HR (95% CI)†
**Tumor-infiltrating lymphocytes (n = 1,470)**							
Negative/low							
Never smoker	458	141	1 (referent)	1 (referent)	262	1 (referent)	1 (referent)
Past smoker	538	158	0.97 (0.77-1.21)	0.92 (0.73-1.16)	320	1.10 (0.94-1.30)	1.03 (0.88-1.22)
Current smoker	104	54	1.94 (1.42-2.66)	1.71 (1.24-2.37)	83	1.71 (1.34-2.19)	2.04 (1.58-2.63)
Intermediate/high							
Never smoker	142	36	1 (referent)	1 (referent)	80	1 (referent)	1 (referent)
Past smoker	187	30	0.60 (0.37-0.97)	0.61 (0.38-0.99)	101	0.96 (0.72-1.29)	0.84 (0.63-1.13)
Current smoker	41	7	0.61 (0.27-1.36)	0.65 (0.29-1.47)	31	1.06 (0.70-1.61)	1.33 (0.87-2.02)
$P_{interaction}$‡			0.004	0.015		0.088	0.11
$P_{interaction}$ (non-current vs. current)§			0.032	0.075		0.079	0.17
**Intratumoral periglandular reaction (n = 1,470)**							
Negative/low							
Never smoker	80	41	1 (referent)	1 (referent)	54	1 (referent)	1 (referent)
Past smoker	90	34	0.70 (0.44-1.10)	0.78 (0.50-1.24)	51	1.04 (0.70-1.52)	0.88 (0.60-1.30)
Current smoker	21	10	0.99 (0.50-1.98)	1.14 (0.56-2.31)	14	1.25 (0.70-2.26)	1.38 (0.76-2.51)
Intermediate/high							
Never smoker	519	136	1 (referent)	1 (referent)	288	1 (referent)	1 (referent)
Past smoker	635	153	0.92 (0.73-1.16)	0.87 (0.69-1.10)	369	1.08 (0.93-1.26)	0.99 (0.85-1.15)
Current smoker	125	52	1.71 (1.24-2.35)	1.50 (1.08-2.09)	101	1.54 (1.23-1.93)	1.83 (1.45-2.31)
$P_{interaction}$‡			0.11	0.42		0.55	0.39
$P_{interaction}$ (non-current vs. current)§			0.27	0.54		0.55	0.46
**Peritumoral lymphocytic reaction (n = 1,465)**							
Negative/low							
Never smoker	92	48	1 (referent)	1 (referent)	66	1 (referent)	1 (referent)
Past smoker	93	43	0.85 (0.56-1.28)	1.13 (0.74-1.71)	58	1.01 (0.71-1.44)	1.02 (0.71-1.45)
Current smoker	24	13	1.08 (0.59-1.99)	1.18 (0.63-2.21)	19	1.18 (0.71-1.97)	1.49 (0.89-2.51)
Intermediate/high							
Never smoker	506	129	1 (referent)	1 (referent)	276	1 (referent)	1 (referent)
Past smoker	628	144	0.90 (0.71-1.14)	0.83 (0.66-1.06)	361	1.10 (0.94-1.28)	0.99 (0.84-1.16)
Current smoker	122	49	1.70 (1.22-2.36)	1.48 (1.06-2.08)	96	1.56 (1.24-1.97)	1.81 (1.43-2.30)
$P_{interaction}$‡			0.26	0.99		0.36	0.59
$P_{interaction}$ (non-current vs. current)§			0.20	0.26		0.39	0.44

**Crohn's-like lymphoid reaction (n = 1,207)**							
Negative/low							
Never smoker	391	131	1 (referent)	1 (referent)	235	1 (referent)	1 (referent)
Past smoker	440	129	0.86 (0.68-1.10)	0.87 (0.68-1.11)	260	1.01 (0.84-1.20)	0.99 (0.83-1.19)
Current smoker	84	43	1.70 (1.21-2.40)	1.50 (1.05-2.14)	67	1.57 (1.20-2.06)	1.96 (1.48-2.59)
Intermediate/high							
Never smoker	98	20	1 (referent)	1 (referent)	53	1 (referent)	1 (referent)
Past smoker	163	21	0.61 (0.33-1.13)	0.58 (0.31-1.08)	83	0.97 (0.69-1.37)	0.84 (0.59-1.19)
Current smoker	31	5	0.70 (0.26-1.87)	0.77 (0.29-2.06)	23	1.08 (0.66-1.77)	1.37 (0.83-2.24)
$P_{interaction}$[‡]			0.088	0.16		0.36	0.34
$P_{interaction}$ (non-current vs. current)[§]			0.17	0.36		0.18	0.34

[*] HRs were estimated for each stratum on the basis of the cases with smoking status at diagnosis, using a re-parameterization of the interaction term in a single regression model for the stratified analyses.

[†] The multivariable Cox regression model initially included age, sex, year of diagnosis, family history of colorectal cancer, body mass index at diagnosis, alcohol consumption at diagnosis, physical activity at diagnosis, processed meat intake at diagnosis, total fiber intake at diagnosis, tumor location, tumor differentiation, AJCC disease stage, microsatellite instability, CpG island methylator phenotype, long interspersed nucleotide element-1 methylation level, *KRAS* mutation, *BRAF* mutation, and *PIK3CA* mutation. A backward elimination with a threshold of $P = 0.05$ was used to select variables in the final models.

[‡] $P_{interaction}$ was calculated using the Wald test for the cross-product of smoking status at diagnosis (ordinal; never, past, and current) and lymphocytic reaction status (binary; negative/low and intermediate/high) in Cox regression model.

[§] $P_{interaction}$ was calculated using the Wald test for the cross-product of smoking status at diagnosis (binary; non-current vs. current) and lymphocytic reaction status (binary; negative/low and intermediate/high) in Cox regression model.

Abbreviations: CI, confidence interval; HR, hazard ratio.

Supplementary table S4. Smoking status at diagnosis and colorectal cancer mortality in strata of levels of lymphocytic reaction patterns

		Colorectal cancer-specific mortality*,†			Overall mortality*,†		
	No. of cases	No. of events	Univariable HR (95% CI)	Multivariable HR (95% CI)‡	No. of events	Univariable HR (95% CI)	Multivariable HR (95% CI)‡
**Tumor-infiltrating lymphocytes**							
**Female (NHS) (n = 842)**							
Negative/low							
Never smoker	257	82	1 (referent)	1 (referent)	151	1 (referent)	1 (referent)
Past smoker	276	80	0.88 (0.64-1.21)	0.82 (0.59-1.13)	165	1.04 (0.80-1.34)	1.01 (0.78-1.30)
Current smoker	69	36	1.65 (1.12-2.43)	1.21 (0.83-1.77)	54	1.57 (1.12-2.19)	1.82 (1.29-2.58)
Intermediate/high							
Never smoker	94	27	1 (referent)	1 (referent)	55	1 (referent)	1 (referent)
Past smoker	112	13	0.41 (0.20-0.84)	0.43 (0.21-0.85)	59	0.93 (0.65-1.33)	0.85 (0.57-1.27)
Current smoker	34	4	0.32 (0.11-0.96)	0.26 (0.08-0.92)	26	0.86 (0.54-1.38)	0.94 (0.52-1.71)
**Male (HPFS) (n = 628)**							
Negative/low							
Never smoker	201	59	1 (referent)	1 (referent)	111	1 (referent)	1 (referent)
Past smoker	262	78	0.94 (0.66-1.33)	0.89 (0.62-1.28)	155	1.06 (0.82-1.38)	0.88 (0.67-1.14)
Current smoker	35	18	2.21 (1.33-3.68)	2.15 (1.21-3.80)	29	1.96 (1.28-2.98)	2.04 (1.34-3.09)
Intermediate/high							
Never smoker	48	9	1 (referent)	1 (referent)	25	1 (referent)	1 (referent)
Past smoker	75	17	0.97 (0.42-2.24)	0.82 (0.34-1.96)	42	1.02 (0.62-1.68)	0.90 (0.54-1.50)
Current smoker	7	3	1.85 (0.42-8.22)	1.37 (0.32-5.80)	5	1.42 (0.52-3.86)	2.07 (0.87-4.89)
**Intratumoral periglandular reaction**							
**Female (NHS) (n = 840)**							
Negative/low							
Never smoker	54	25	1 (referent)	1 (referent)	34	1 (referent)	1 (referent)
Past smoker	47	12	0.48 (0.24-0.97)	0.57 (0.28-1.19)	24	1.11 (0.60-2.03)	1.00 (0.58-1.71)
Current smoker	15	7	1.18 (0.50-2.81)	0.80 (0.39-1.65)	10	1.74 (0.76-3.96)	1.47 (0.65-3.32)
Intermediate/high							
Never smoker	295	84	1 (referent)	1 (referent)	172	1 (referent)	1 (referent)
Past smoker	340	80	0.82 (0.59-1.13)	0.76 (0.54-1.06)	199	0.99 (0.79-1.24)	0.95 (0.75-1.19)
Current smoker	89	34	1.27 (0.85-1.89)	0.98 (0.63-1.50)	71	1.26 (0.95-1.69)	1.42 (1.01-2.00)
**Male (HPFS) (n = 630)**							
Negative/low							
Never smoker	26	16	1 (referent)	1 (referent)	20	1 (referent)	1 (referent)
Past smoker	43	22	0.60 (0.32-1.12)	0.74 (0.39-1.40)	27	0.57 (0.32-1.02)	0.63 (0.33-1.20)
Current smoker	6	3	0.71 (0.16-3.20)	3.85 (1.18-12.6)	4	0.67 (0.18-2.43)	1.34 (0.37-4.90)
Intermediate/high							
Never smoker	224	52	1 (referent)	1 (referent)	116	1 (referent)	1 (referent)
Past smoker	295	73	0.99 (0.69-1.43)	0.92 (0.62-1.35)	170	1.11 (0.87-1.43)	0.92 (0.72-1.17)
Current smoker	36	18	2.69 (1.58-4.61)	2.21 (1.25-3.90)	30	2.08 (1.36-3.17)	2.02 (1.34-3.05)

**Peritumoral lymphocytic reaction**							
**Female (NHS) (n = 838)**							
Negative/low							
Never smoker	51	23	1 (referent)	1 (referent)	34	1 (referent)	1 (referent)
Past smoker	51	18	0.70 (0.37-1.31)	1.20 (0.61-2.37)	28	1.14 (0.59-2.17)	1.19 (0.70-2.05)
Current smoker	17	7	0.95 (0.38-2.35)	0.89 (0.41-1.92)	12	1.45 (0.71-2.96)	1.76 (0.83-3.72)
Intermediate/high							
Never smoker	297	86	1 (referent)	1 (referent)	172	1 (referent)	1 (referent)
Past smoker	335	74	0.76 (0.55-1.05)	0.66 (0.48-0.92)	195	0.98 (0.79-1.23)	0.92 (0.73-1.16)
Current smoker	87	34	1.30 (0.88-1.93)	0.94 (0.61-1.43)	69	1.29 (0.95-1.74)	1.37 (0.97-1.93)
**Male (HPFS) (n = 627)**							
Negative/low	41	25	1 (referent)	1 (referent)	32	1 (referent)	1 (referent)
Never smoker	42	25	0.82 (0.46-1.47)	1.05 (0.55-2.00)	30	0.74 (0.43-1.27)	0.87 (0.49-1.57)
Past smoker	7	6	1.50 (0.67-3.33)	1.15 (0.34-3.93)	7	1.41 (0.67-2.97)	1.10 (0.44-2.74)
Current smoker							
Intermediate/high							
Never smoker	209	43	1 (referent)	1 (referent)	104	1 (referent)	1 (referent)
Past smoker	293	70	1.10 (0.74-1.62)	0.98 (0.65-1.47)	166	1.18 (0.91-1.53)	0.96 (0.75-1.24)
Current smoker	35	15	2.51 (1.36-4.63)	2.49 (1.34-4.64)	27	1.97 (1.26-3.09)	2.09 (1.34-3.25)
**Crohn's-like lymphoid reaction**							
**Female (NHS) (n = 701)**							
Negative/low							
Never smoker	229	74	1 (referent)	1 (referent)	138	1 (referent)	1 (referent)
Past smoker	246	64	0.75 (0.53-1.06)	0.77 (0.54-1.11)	142	0.92 (0.69-1.21)	0.93 (0.71-1.22)
Current smoker	58	29	1.56 (1.03-2.36)	1.16 (0.75-1.81)	45	1.38 (0.93-2.07)	1.64 (1.06-2.53)
Intermediate/high							
Never smoker	60	16	1 (referent)	1 (referent)	36	1 (referent)	1 (referent)
Past smoker	85	8	0.31 (0.13-0.77)	0.29 (0.11-0.77)	45	0.92 (0.61-1.39)	0.85 (0.56-1.31)
Current smoker	23	2	0.32 (0.07-1.53)	0.46 (0.13-1.67)	18	0.99 (0.63-1.56)	1.54 (0.91-1.62)
**Male (HPFS) (n = 506)**							
Negative/low							
Never smoker	162	57	1 (referent)	1 (referent)	97	1 (referent)	1 (referent)
Past smoker	194	65	0.93 (0.64-1.34)	0.91 (0.62-1.33)	118	1.04 (0.78-1.39)	0.95 (0.71-1.27)
Current smoker	26	14	1.95 (1.13-3.36)	2.21 (1.29-3.81)	22	1.77 (1.09-2.86)	2.06 (1.29-3.27)
Intermediate/high							
Never smoker	38	4	1 (referent)	1 (referent)	17	1 (referent)	1 (referent)
Past smoker	78	13	0.97 (0.29-3.27)	0.80 (0.24-2.73)	38	0.93 (0.52-1.69)	0.67 (0.40-1.14)
Current smoker	8	3	2.07 (0.41-10.5)	1.89 (0.38-9.54)	5	1.45 (0.61-3.43)	1.11 (0.44-2.78)

* IPW was applied to reduce a bias due to the availability of tumor tissue after cancer diagnosis (see "Statistical Analysis" subsection for details).

† HRs were estimated for each stratum on the basis of the cases with smoking status at diagnosis, using a re-parameterization of the interaction term in a single regression model for the stratified analyses.

‡ The multivariable Cox regression model initially included age, year of diagnosis, family history of colorectal cancer, body mass index at diagnosis, alcohol consumption at diagnosis, physical activity at diagnosis, processed meat intake at diagnosis, total fiber intake at diagnosis, tumor location, tumor differentiation, AJCC disease stage, microsatellite instability, CpG island methylator phenotype, long interspersed nucleotide element-1 methylation level, *KRAS* mutation, *BRAF* mutation, and *PIK3CA* mutation. A backward elimination with a threshold of $P = 0.05$ was used to select variables in the final models.

Abbreviations: CI, confidence interval; HPFS, Health Professional Follow-up Study, HR, hazard ratio; IPW, inverse probability weighting; NHS, Nurses' Health Study.

Supplementary table S5. Smoking status at diagnosis and colorectal cancer mortality in strata of levels of lymphocytic reaction patterns

		Colorectal cancer-specific mortality*,†			Overall mortality*,†		
	No. of cases	No. of events	Univariable HR (95% CI)	Multivariable HR (95% CI)‡	No. of events	Univariable HR (95% CI)	Multivariable HR (95% CI)‡
**Tumor-infiltrating lymphocytes**							
**Colon (n = 1,157)**							
Negative/low							
Never smoker	354	107	1 (referent)	1 (referent)	202	1 (referent)	1 (referent)
Past smoker	409	125	1.04 (0.79-1.36)	0.94 (0.71-1.24)	246	1.17 (0.94-1.45)	1.05 (0.85-1.29)
Current smoker	68	32	1.59 (1.05-2.39)	1.40 (0.94-2.10)	53	1.53 (1.09-2.14)	1.74 (1.24-2.43)
Intermediate/high							
Never smoker	127	32	1 (referent)	1 (referent)	73	1 (referent)	1 (referent)
Past smoker	161	24	0.49 (0.28-0.87)	0.52 (0.29-0.90)	89	0.95 (0.70-1.30)	0.85 (0.61-1.19)
Current smoker	38	6	0.46 (0.18-1.16)	0.38 (0.13-1.10)	29	0.96 (0.62-1.48)	0.98 (0.56-1.73)
**Rectum (n = 308)**							
Negative/low							
Never smoker	103	33	1 (referent)	1 (referent)	59	1 (referent)	1 (referent)
Past smoker	127	33	0.59 (0.36-0.99)	0.58 (0.34-0.99)	77	0.88 (0.60-1.30)	0.74 (0.49-1.10)
Current smoker	35	21	2.13 (1.26-3.61)	1.58 (0.86-2.91)	29	1.96 (1.21-3.19)	1.99 (1.11-3.56)
Intermediate/high							
Never smoker	15	4	1 (referent)	1 (referent)	7	1 (referent)	1 (referent)
Past smoker	25	5	0.51 (0.13-1.96)	0.71 (0.14-3.73)	11	0.85 (0.34-2.15)	0.96 (0.37-2.50)
Current smoker	3	1	0.63 (0.06-6.19)	0.72 (0.09-5.74)	2	0.57 (0.09-3.74)	0.95 (0.21-4.34)
**Intratumoral periglandular reaction**							
**Colon (n = 1,156)**							
Negative/low							
Never smoker	61	32	1 (referent)	1 (referent)	42	1 (referent)	1 (referent)
Past smoker	74	27	0.67 (0.39-1.15)	0.66 (0.39-1.12)	41	1.32 (0.73-2.37)	1.04 (0.63-1.72)
Current smoker	16	7	0.94 (0.37-2.38)	0.82 (0.35-1.91)	10	1.43 (0.59-3.50)	1.28 (0.52-3.10)
Intermediate/high							
Never smoker	418	107	1 (referent)	1 (referent)	233	1 (referent)	1 (referent)
Past smoker	496	122	0.95 (0.72-1.25)	0.88 (0.67-1.17)	291	1.09 (0.90-1.31)	0.99 (0.82-1.19)
Current smoker	91	32	1.26 (0.83-1.90)	1.14 (0.74-1.75)	73	1.30 (0.99-1.71)	1.42 (1.02-1.97)
**Rectum (n = 309)**							
Negative/low							
Never smoker	18	8	1 (referent)	1 (referent)	11	1 (referent)	1 (referent)
Past smoker	15	7	0.80 (0.30-2.11)	0.93 (0.41-2.10)	10	1.14 (0.43-3.05)	1.06 (0.46-2.48)
Current smoker	5	3	1.48 (0.53-4.08)	1.10 (0.32-3.79)	4	2.03 (0.73-5.69)	2.22 (0.79-6.26)
Intermediate/high							
Never smoker	101	29	1 (referent)	1 (referent)	55	1 (referent)	1 (referent)

Past smoker	137	30	0.57 (0.33-0.99)	0.53 (0.28-0.97)	77	0.87 (0.59-1.27)	0.71 (0.47-1.08)
Current smoker	33	19	2.20 (1.21-4.00)	1.55 (0.80-3.00)	27	1.76 (1.01-3.05)	1.78 (0.97-3.29)
**Peritumoral lymphocytic reaction**							
**Colon (n = 1,152)**							
Negative/low							
Never smoker	72	39	1 (referent)	1 (referent)	53	1 (referent)	1 (referent)
Past smoker	70	34	0.90 (0.55-1.47)	1.18 (0.69-2.02)	45	1.44 (0.83-2.52)	1.36 (0.85-2.18)
Current smoker	19	9	0.80 (0.33-1.91)	0.96 (0.42-2.16)	14	1.16 (0.56-2.38)	1.59 (0.75-3.35)
Intermediate/high							
Never smoker	406	100	1 (referent)	1 (referent)	222	1 (referent)	1 (referent)
Past smoker	497	115	0.92 (0.69-1.22)	0.83 (0.62-1.10)	286	1.09 (0.90-1.31)	0.97 (0.80-1.17)
Current smoker	88	30	1.31 (0.86-1.98)	1.07 (0.68-1.67)	69	1.34 (1.01-1.78)	1.36 (0.97-1.90)
**Rectum (n = 308)**							
Negative/low							
Never smoker	19	8	1 (referent)	1 (referent)	12	1 (referent)	1 (referent)
Past smoker	22	9	0.65 (0.24-1.75)	0.91 (0.36-2.25)	13	0.71 (0.23-2.17)	0.79 (0.34-1.83)
Current smoker	4	3	1.97 (0.68-5.67)	1.08 (0.30-3.87)	4	2.64 (0.95-7.32)	1.95 (0.66-5.76)
Intermediate/high							
Never smoker	100	29	1 (referent)	1 (referent)	54	1 (referent)	1 (referent)
Past smoker	129	28	0.55 (0.32-0.96)	0.51 (0.28-0.94)	74	0.90 (0.61-1.31)	0.75 (0.49-1.14)
Current smoker	34	19	2.08 (1.15-3.76)	1.56 (0.80-3.02)	27	1.71 (0.99-2.96)	1.84 (1.00-3.41)
**Crohn's-like lymphoid reaction**							
**Colon (n = 956)**							
Negative/low	311	103	1 (referent)	1 (referent)	185	1 (referent)	1 (referent)
Never smoker	328	99	0.93 (0.70-1.25)	0.95 (0.72-1.27)	191	1.07 (0.84-1.36)	1.06 (0.85-1.32)
Past smoker	55	24	1.40 (0.89-2.19)	1.24 (0.78-1.97)	42	1.33 (0.88-2.00)	1.47 (0.94-2.30)
Current smoker							
Intermediate/high							
Never smoker	89	17	1 (referent)	1 (referent)	49	1 (referent)	1 (referent)
Past smoker	143	16	0.44 (0.21-0.90)	0.38 (0.18-0.81)	75	0.96 (0.68-1.36)	0.78 (0.54-1.12)
Current smoker	30	4	0.51 (0.15-1.72)	0.62 (0.19-2.03)	22	1.07 (0.71-1.61)	1.34 (0.83-2.18)
**Rectum (n = 247)**							
Negative/low							
Never smoker	80	28	1 (referent)	1 (referent)	50	1 (referent)	1 (referent)
Past smoker	110	30	0.54 (0.31-0.93)	0.51 (0.29-0.90)	69	0.78 (0.51-1.20)	0.70 (0.47-1.05)
Current smoker	28	18	1.90 (1.05-3.45)	1.66 (0.84-3.27)	24	1.63 (0.91-2.92)	2.09 (1.14-3.81)
Intermediate/high							
Never smoker	8	2	1 (referent)	1 (referent)	3	1 (referent)	1 (referent)
Past smoker	20	5	1.04 (0.22-4.91)	1.14 (0.25-5.20)	8	1.20 (0.33-4.33)	1.31 (0.31-5.46)
Current smoker	1	1	4.52 (1.28-15.9)	1.61 (0.43-6.05)	1	3.71 (1.32-10.4)	1.22 (0.35-4.20)

* IPW was applied to reduce a bias due to the availability of tumor tissue after cancer diagnosis (see "Statistical Analysis" subsection for details).

† HRs were estimated for each stratum on the basis of the cases with smoking status at diagnosis, using a re-parameterization of the interaction term in a single regression model for the stratified analyses.

‡ The multivariable Cox regression model initially included age, sex, year of diagnosis, family history of colorectal cancer, body mass index at diagnosis, alcohol consumption at diagnosis, physical activity at diagnosis, processed meat intake at diagnosis, total fiber intake at diagnosis, tumor differentiation, AJCC disease stage, microsatellite instability, CpG island methylator phenotype, long interspersed nucleotide element-1 methylation level, *KRAS* mutation, *BRAF* mutation, and *PIK3CA* mutation. A backward elimination with a threshold of P = 0.05 was used to select variables in the final models.

Abbreviations: CI, confidence interval; HR, hazard ratio; IPW, inverse probability weighting.

Supplementary table S6. Duration of smoking cessation years at diagnosis and colorectal cancer mortality in strata of levels of lymphocytic reaction patterns

		Colorectal cancer-specific mortality[*,†]			Overall mortality[*,†]		
	No. of cases	No. of events	Univariable HR (95% CI)	Multivariable HR (95% CI)[‡]	No. of events	Univariable HR (95% CI)	Multivariable HR (95% CI)[‡]
**Tumor-infiltrating lymphocytes (n = 1,391)**							
Negative/low							
Never smoker	426	124	1 (referent)	1 (referent)	238	1 (referent)	1 (referent)
Past smoker ≥ 10 years quit	277	81	0.92 (0.68-1.25)	0.83 (0.60-1.15)	158	1.02 (0.81-1.29)	0.88 (0.70-1.12)
Past smoker < 10 years quit	228	62	0.87 (0.64-1.20)	0.78 (0.57-1.06)	136	1.10 (0.85-1.41)	1.04 (0.82-1.31)
Current smoker	104	54	1.88 (1.36-2.60)	1.50 (1.08-2.08)	83	1.71 (1.29-2.26)	2.02 (1.53-2.68)
Intermediate/high							
Never smoker	135	35	1 (referent)	1 (referent)	76	1 (referent)	1 (referent)
Past smoker ≥ 10 years quit	85	16	0.56 (0.29-1.09)	0.57 (0.28-1.16)	47	0.92 (0.63-1.33)	0.78 (0.53-1.14)
Past smoker < 10 years quit	95	13	0.50 (0.25-0.99)	0.52 (0.26-1.06)	51	1.02 (0.72-1.43)	0.94 (0.66-1.35)
Current smoker	41	7	0.46 (0.20-1.11)	0.40 (0.15-1.07)	31	0.93 (0.60-1.43)	1.09 (0.68-1.76)
$P_{interaction}$[§]			0.003	0.010		0.065	0.082
**Intratumoral periglandular reaction (n = 1,390)**							
Negative/low							
Never smoker	74	36	1 (referent)	1 (referent)	49	1 (referent)	1 (referent)
Past smoker ≥ 10 years quit	34	16	0.82 (0.45-1.49)	0.88 (0.47-1.67)	18	1.01 (0.53-1.91)	0.80 (0.45-1.44)
Past smoker < 10 years quit	49	13	0.47 (0.24-0.92)	0.56 (0.29-1.10)	27	1.15 (0.66-2.01)	1.01 (0.61-1.66)
Current smoker	21	10	1.10 (0.51-2.38)	0.88 (0.41-1.88)	14	1.52 (0.72-3.21)	1.28 (0.58-2.81)
Intermediate/high							
Never smoker	485	123	1 (referent)	1 (referent)	265	1 (referent)	1 (referent)
Past smoker ≥ 10 years quit	328	80	0.87 (0.64-1.19)	0.76 (0.55-1.06)	186	1.01 (0.82-1.23)	0.86 (0.69-1.07)
Past smoker < 10 years quit	274	62	0.86 (0.62-1.18)	0.78 (0.57-1.08)	160	1.08 (0.86-1.34)	1.07 (0.86-1.33)
Current smoker	125	52	1.58 (1.13-2.21)	1.26 (0.88-1.80)	101	1.45 (1.13-1.86)	1.63 (1.25-2.14)
$P_{interaction}$[§]			0.18	0.29		0.87	0.59

**Peritumoral lymphocytic reaction (n = 1,385)**							
Negative/low							
Never smoker	83	42	1 (referent)	1 (referent)	59	1 (referent)	1 (referent)
Past smoker ≥ 10 years quit	38	21	1.03 (0.59-1.78)	1.38 (0.75-2.56)	26	1.40 (0.78-2.50)	1.15 (0.70-1.91)
Past smoker < 10 years quit	49	17	0.60 (0.33-1.08)	0.78 (0.42-1.45)	26	0.87 (0.47-1.64)	0.80 (0.49-1.31)
Current smoker	24	13	1.04 (0.52-2.10)	0.89 (0.47-1.68)	19	1.34 (0.72-2.51)	1.28 (0.70-2.34)
Intermediate/high							
Never smoker	475	117	1 (referent)	1 (referent)	255	1 (referent)	1 (referent)
Past smoker ≥ 10 years quit	320	75	0.85 (0.62-1.16)	0.72 (0.52-1.01)	177	0.98 (0.80-1.21)	0.80 (0.64-1.00)
Past smoker < 10 years quit	274	58	0.82 (0.59-1.14)	0.73 (0.53-1.02)	161	1.12 (0.90-1.39)	1.08 (0.87-1.36)
Current smoker	122	49	1.58 (1.12-2.22)	1.23 (0.85-1.80)	96	1.46 (1.13-1.89)	1.56 (1.17-2.09)
$P_{interaction}$[§]			0.25	0.55		0.61	0.47
**Crohn's-like lymphoid reaction (n = 1,143)**							
Negative/low							
Never smoker	363	115	1 (referent)	1 (referent)	215	1 (referent)	1 (referent)
Past smoker ≥ 10 years quit	219	67	0.89 (0.64-1.22)	0.86 (0.61-1.21)	124	0.93 (0.72-1.22)	0.83 (0.64-1.08)
Past smoker < 10 years quit	196	49	0.73 (0.52-1.04)	0.74 (0.52-1.05)	115	0.99 (0.76-1.30)	1.06 (0.82-1.37)
Current smoker	84	43	1.72 (1.22-2.44)	1.34 (0.92-1.94)	67	1.51 (1.08-2.10)	1.75 (1.24-2.49)
Intermediate/high							
Never smoker	95	20	1 (referent)	1 (referent)	51	1 (referent)	1 (referent)
Past smoker ≥ 10 years quit	75	8	0.30 (0.13-0.71)	0.26 (0.10-0.67)	36	0.77 (0.52-1.12)	0.55 (0.37-0.82)
Past smoker < 10 years quit	80	11	0.55 (0.26-1.20)	0.57 (0.26-1.25)	44	1.14 (0.78-1.67)	1.06 (0.72-1.55)
Current smoker	31	5	0.54 (0.18-1.59)	0.61 (0.22-1.73)	23	1.10 (0.74-1.64)	1.27 (0.81-1.99)
$P_{interaction}$[§]			0.093	0.23		0.76	0.82

[*] IPW was applied to reduce a bias due to the availability of tumor tissue after cancer diagnosis (see "Statistical Analysis" subsection for details).

[†] HRs were estimated for each stratum on the basis of the cases with duration of smoking cessation years at diagnosis, using a re-parameterization of the interaction term in a single regression model for the stratified analyses.

[‡] The multivariable Cox regression model initially included age, sex, year of diagnosis, family history of colorectal cancer, body mass index at diagnosis, alcohol consumption at diagnosis, physical activity at diagnosis, processed meat intake at diagnosis, total fiber intake at diagnosis, tumor location, tumor differentiation, AJCC disease stage, microsatellite instability, CpG island methylator phenotype, long interspersed nucleotide element-1 methylation level, *KRAS* mutation, *BRAF* mutation, and *PIK3CA* mutation. A backward elimination with a threshold of $P = 0.05$ was used to select variables in the final models.

[§] $P_{interaction}$ was calculated using the Wald test for the cross-product of duration of smoking cessation years at diagnosis (ordinal; non-current, ≥ 10 years, < 10 years, and current) and lymphocytic reaction status (binary; negative/low and intermediate/high) in Cox regression model.

Abbreviations: IPW, inverse probability weighting; CI, confidence interval; HR, hazard ratio.

Supplementary table S7. Post diagnostic smoking status and colorectal cancer mortality in strata of levels of lymphocytic reaction patterns

	Postdiagnosis smoking status*	No. of cases	Colorectal cancer-specific mortality†,‡			Overall mortality†,‡		
			No. of events	Univariable HR (95% CI)	Multivariable HR (95% CI)§	No. of events	Univariable HR (95% CI)	Multivariable HR (95% CI)§
**Tumor-infiltrating lymphocytes (n = 1,289)**								
**Negative/low**								
	Non-current	904	216	1 (referent)	1 (referent)	492	1 (referent)	1 (referent)
	Current	53	20	1.93 (1.18-3.15)	1.77 (1.09-2.88)	42	1.89 (1.37-2.61)	1.92 (1.37-2.70)
**Intermediate/high**								
	Non-current	307	44	1 (referent)	1 (referent)	161	1 (referent)	1 (referent)
	Current	25	5	1.13 (0.42-3.05)	1.33 (0.47-3.80)	18	0.98 (0.57-1.68)	1.34 (0.82-2.19)
**Intratumoral periglandular reaction (n = 1,289)**								
**Negative/low**								
	Non-current	142	46	1 (referent)	1 (referent)	75	1 (referent)	1 (referent)
	Current	8	1	0.27 (0.04-1.85)	0.24 (0.04-1.56)	4	0.85 (0.32-2.24)	0.67 (0.23-2.00)
**Intermediate/high**								
	Non-current	1069	214	1 (referent)	1 (referent)	578	1 (referent)	1 (referent)
	Current	70	24	2.01 (1.27-3.19)	1.99 (1.27-3.14)	56	1.65 (1.22-2.29)	1.94 (1.43-2.62)
**Peritumoral lymphocytic reaction (n = 1,285)**								
**Negative/low**								
	Non-current	149	58	1 (referent)	1 (referent)	89	1 (referent)	1 (referent)
	Current	10	3	0.36 (0.10-1.44)	0.40 (0.10-1.53)	8	0.91 (0.50-1.63)	1.06 (0.51-2.23)
**Intermediate/high**								
	Non-current	1058	202	1 (referent)	1 (referent)	564	1 (referent)	1 (referent)
	Current	68	22	2.14 (1.34-3.41)	2.00 (1.26-3.18)	52	1.70 (1.24-2.35)	1.87 (1.36-2.57)
**Crohn's-like lymphoid reaction (n = 1,063)**								
**Negative/low**								
	Non-current	742	182	1 (referent)	1 (referent)	413	1 (referent)	1 (referent)
	Current	44	19	2.17 (1.34-3.54)	2.01 (1.28-3.16)	34	1.74 (1.34-2.66)	1.85 (1.22-2.79)
**Intermediate/high**								
	Non-current	254	32	1 (referent)	1 (referent)	128	1 (referent)	1 (referent)
	Current	23	5	1.74 (0.59-5.12)	2.02 (0.74-6.36)	18	1.44 (0.98-2.12)	1.67 (1.01-2.76)

[*] Postdiagnosis smoking status was derived from the available questionnaire at least more than 6 months after colorectal cancer diagnosis.

[†] IPW was applied to reduce a bias due to the availability of tumor tissue after cancer diagnosis (see "Statistical Analysis" subsection for details).

[‡] HRs were estimated for each stratum on the basis of the cases with smoking status at diagnosis, using a re-parameterization of the interaction term in a single regression model for the stratified analyses.

[§] The multivariable Cox regression model initially included age, sex, year of diagnosis, family history of colorectal cancer, body mass index at diagnosis, alcohol consumption at diagnosis, physical activity at diagnosis, processed meat intake at diagnosis, total fiber intake at diagnosis, cumulative pack-years of cigarettes, tumor location, tumor differentiation, AJCC disease stage, microsatellite instability, CpG island methylator phenotype, long interspersed nucleotide element-1 methylation level, *KRAS* mutation, *BRAF* mutation, and *PIK3CA* mutation. A backward elimination with a threshold of $P = 0.05$ was used to select variables in the final models.

Abbreviations: CI, confidence interval; HR, hazard ratio; IPW, inverse probability weighting.

Supplementary table S8. Prediction models for colorectal cancer-specific survival and overall survival

	AUC	*P*-value[§]
**Five-year colorectal cancer-specific survival**		
Baseline[*,†]	0.8128	-
Baseline + smoking status at diagnosis	0.8128	0.85
Baseline + TIL status	0.8137	0.73
Baseline + smoking status at diagnosis + TIL status	0.8137	0.74
**Five-year overall survival**		
Baseline[*,‡]	0.7603	-
Baseline + smoking status at diagnosis	0.7614	0.32
Baseline + TIL status	0.7631	0.30
Baseline + smoking status at diagnosis + TIL status	0.7643	0.17

[*] The multivariable logistic regression model initially included age, sex, year of diagnosis, family history of colorectal cancer, body mass index at diagnosis, alcohol consumption at diagnosis, physical activity at diagnosis, processed meat intake at diagnosis, total fiber intake at diagnosis, tumor location, tumor differentiation, AJCC disease stage, microsatellite instability, CpG island methylator phenotype, long interspersed nucleotide element-1 methylation level, *KRAS* mutation, *BRAF* mutation, and *PIK3CA* mutation. A backward elimination with a threshold of *P* = 0.05 was used to select variables in the final models.

[†] Baseline model included age, year of diagnosis, tumor location, tumor differentiation, AJCC stage, microsatellite instability, long interspersed nucleotide element-1 methylation level, *KRAS* mutation, and *BRAF* mutation.

[‡] Baseline model included age, year of diagnosis, tumor differentiation, AJCC disease stage, microsatellite instability, and *BRAF* mutation.

[§] The Mann-Whitney U test was performed to compare the baseline model of area under curve.

Abbreviations: AUC, area under curve; TIL tumor-infiltrating lymphocytes.
